# Loss of Lipid Carrier ApoE Exacerbates Brain Glial and Inflammatory Responses after Lysosomal GBA1 Inhibition

**DOI:** 10.3390/cells12212564

**Published:** 2023-11-02

**Authors:** Kyle J. Connolly, Juliette Margaria, Erika Di Biase, Oliver Cooper, Penelope J. Hallett, Ole Isacson

**Affiliations:** Departments of Psychiatry and Neurology Harvard Medical School, Neuroregeneration Institute, McLean Hospital, Belmont, MA 02478, USA

**Keywords:** Parkinson’s disease, Lewy body dementia, Alzheimer’s disease, APOE, GBA1, inflammation, lipids, cholesterol, lysosomes, glia

## Abstract

Tightly regulated and highly adaptive lipid metabolic and transport pathways are critical to maintaining brain cellular lipid homeostasis and responding to lipid and inflammatory stress to preserve brain function and health. Deficits in the lipid handling genes *APOE* and *GBA1* are the most significant genetic risk factors for Lewy body dementia and related dementia syndromes. Parkinson’s disease patients who carry both APOE4 and GBA1 variants have accelerated cognitive decline compared to single variant carriers. To investigate functional interactions between brain ApoE and GBA1, in vivo GBA1 inhibition was tested in WT versus ApoE-deficient mice. The experiments demonstrated glycolipid stress caused by GBA1 inhibition in WT mice induced ApoE expression in several brain regions associated with movement and dementia disorders. The absence of ApoE in ApoE-KO mice amplified complement C1q elevations, reactive microgliosis and astrocytosis after glycolipid stress. Mechanistically, GBA1 inhibition triggered increases in cell surface and intracellular lipid transporters ABCA1 and NPC1, respectively. Interestingly, the absence of NPC1 in mice also triggered elevations of brain ApoE levels. These new data show that brain ApoE, GBA1 and NPC1 functions are interconnected in vivo, and that the removal or reduction of ApoE would likely be detrimental to brain function. These results provide important insights into brain ApoE adaptive responses to increased lipid loads.

## 1. Introduction

Clinical and genetic evidence clearly show that variants in ApoE and GBA1 are the highest risk factors for Lewy body dementia (LBD) and dementia onset in Parkinson’s disease (PD) patients [1,2]. Furthermore, cognitive decline is accelerated in PD patients who carry both *APOE4* and *GBA1* variants compared to single variant carriers [1]. Interestingly, gene expression studies at the cellular level in an ApoE3 variant human carrier brain show that high ApoE gene expression coincides with less regional brain tau pathology in autosomal dominant Alzheimer’s disease [3]. These genetic findings indicate that the important functions of ApoE and GBA1 in the brain could be linked. ApoE is the most abundant brain apolipoprotein and its primary function is to exchange lipids between neuronal and glial cell types [4,5]. This lipid exchange is critical to maintaining the homeostatic balance of cellular lipids and is increased during physiological adaptations to lipid, inflammatory and cellular stresses [4,6,7,8,9,10]. Brain ApoE is primarily synthesized by astrocytes under homeostatic conditions [11,12], whereas increased microglial and neuronal ApoE production is triggered during inflammation, varied lipid stresses and neuronal damage [13,14,15,16]. The fact that brain cell type-specific and total ApoE levels correlate with multiple diverse stress conditions and damage in the brain, indicates that ApoE-mediated lipid transport and mechanisms for inflammatory activation and resolution are functionally integrated [10,17,18].

Deficits in lipid metabolism coupled with disrupted or insufficient lipid transport cause lipid abnormalities and excessive lipid storage [18,19,20], triggering downstream cellular inflammatory activations, mitochondrial dysfunction, oxidative stress and protein aggregation [21,22]. We hypothesize that changes in brain ApoE production reflect context-dependent and integrated cellular adaptations in response to lipid transport demands that occur during aging and brain lipid elevations associated with aging and LBD.

Previous experiments have demonstrated that sustained systemic GBA1 inhibition in mice causes the accumulation of glycolipid substrates in the brain [18,21]. During cellular lipid challenges, cells must engage appropriate lipid processing and trafficking pathways to adapt to lipid elevations and maintain homeostasis [7,9]. ApoE is the major brain apolipoprotein [23,24] that binds lipids and facilitates extracellular lipid transport between cells [25,26]. It is likely that ApoE-lipid transport is central to the lipid biological adaptations during cellular lipid elevations [4,7,9,18,27].

Mouse models expressing the human ApoE4 variant have produced sometimes conflicting results, which has led to confusion in the field of whether the elimination or improved function of ApoE4 would be of translational value. Human ApoE2/3/4 variants only differ by single amino acid substitutions at positions 112 and 158. Interestingly, both the human ApoE4 variant and endogenous rodent ApoE carry two arginine residues, suggesting that rodent ApoE closely resembles the human ApoE4 variant [28]. From an experimental perspective, the knockout of ApoE may therefore be a preferred model with which to investigate the functional and physiological changes that occur in humans carrying ApoE4. For these reasons, the current study utilized mice deficient in ApoE to model the most likely outcome of the human ApoE4 variant.

Given the clear role of ApoE variants and GBA in PD and LBD, the work presented here determined the role of ApoE in situations of glycolipid stress in the brain using in vivo paradigms of sustained systemic GBA1 inhibition, followed by paradigms of glycolipid stress in mice lacking the ApoE gene. Using cell-specific markers for microglia, astrocytes and neurons, the ApoE responses were tested in PD- and LBD-vulnerable brain regions of the cortex, hippocampus and substantia nigra. Further biochemical analysis of relevant pathway proteins involved in cellular lipid transport and lysosomal responses to lipid stress revealed functional connections between ApoE and the lysosomal lipid transporter NPC1. The results revealed that brain ApoE function is increased during glycolipid stress caused by GBA1 inhibition. The absence of ApoE amplified specific brain regional patterns of microglial and astrocytic responses to glycolipid stress, involving integrated cellular biochemical and inflammatory changes that are likely triggered by increased lipid transport demands, similar to those occurring during human brain lipid elevations associated with PD and LBD. Overall, the findings reveal that ApoE function is involved in responses to elevated glycolipid levels caused by GBA1 loss of function. The results also point to functional interconnections for lipid and cholesterol transport at the intracellular lysosomal level, evident by similar increases in ApoE, and complement C1q levels seen in *NPC1*^−/−^ mice, which have cytoplasmic glycolipid and cholesterol accumulation.

## 2. Materials and Methods

### 2.1. Animals

Male C57BL/6J (WT) mice (strain #000664) and homozygous *Apoe^tm1Unc^* (ApoE-KO) mice (strain #002052) obtained from Jackson Laboratory, Maine, USA, were used at 3 months of age. Animals were raised in standard conditions in a 12-h dark 12-h light cycle with ad libitum access to food and water. All animal experiments were performed in accordance with current National Institute of Health guidelines and were approved by the Institutional Animal Care and Use Committee (IACUC) at McLean Hospital, Harvard Medical School. Brain samples from BALB/cNctr-*Npc1^m1N^*/J (*NPC1^−/−^*) mice [29,30,31] were provided by Dr Frances Platt (University of Oxford).

### 2.2. Systemic Inhibition of Lysosomal GBA1 Enzyme in Mice

Mice received an intraperitoneal (i.p.) injection of 5 μL/g body weight with 20 mg/mL GBA1 inhibitor conduritol-β-epoxide (CBE, EMD Millipore) (100 mg/kg) or a vehicle for 18 consecutive days. Injectable solutions were prepared in sterile 0.9% saline solution (Vedco, St Joseph, MO, USA) containing 10% DMSO. Mice were euthanized 24 h following the final injection.

### 2.3. GBA1 Activity Assay

Lysosomal GBA1 activity was measured in protein extracts prepared from the cerebral cortex in GBA1 activity sample diluent, as previously described [19,21,22,32].

### 2.4. Immunofluorescence and Immunohistochemistry

Mice were terminally anesthetized by i.p. injection with sodium pentobarbital and transcardially perfused with cold 0.2 M phosphate buffer, pH 7.4, containing 0.9% saline and 0.1% heparin, followed by cold 4% paraformaldehyde in phosphate buffer. Brains were extracted and immersed in 4% paraformaldehyde for 24 h at 4 °C, then placed in 30% sucrose for 24 h at 4 °C. Brains were sectioned into 40-μm thick coronal slices on a sliding microtome (Microm HM 450, Thermo Scientific, Waltham, MA, USA) and stored at −20 °C in antifreeze solution (30% glycerol, 30% ethoxyethanol, 40% PBS) until use.

For immunofluorescence, heat-induced epitope retrieval was performed by incubating free-floating brain slices in pH 9 Tris-EDTA solution (Dako, Glostrup, Denmark) or pH 6 citrate solution (Dako) pre-heated to 90 °C for 20 min with gentle rocking. Sections were blocked for 1 h using 10% normal serum in PBS with 0.3% Triton X-100 (PBSTr) or, for mouse primary antibodies, using Mouse on Mouse blocking solution (Vector Laboratories, Burlingame, CA, USA). Slices were incubated overnight at room temperature with primary antibodies against the following target proteins: ApoE (Abcam, Waltham, MA, USA, ab183579, 1:1,000), C1q (Abcam, ab182451, 1:250), GFAP (EMD Millipore, Burlington, MA, USA, MAB360, 1:1,000), Iba1 (Novus, Centennial, CO, USA, NB100-1028, 1:500), LAMP1 (Abcam, ab208943, 1:100), TUJ1 (Covance, MMS-435P, 1:1000). Slices were washed twice in PBSTr for 15 min and then incubated with Alexa Fluor-conjugated secondary antibodies for 1 h. Nuclei were stained with 5 μg/mL Hoechst 33342 (Fisher Scientific, Waltham, MA, USA) for 10 min. Brain sections were mounted on slides and coverslipped in ProLong Diamond medium. Confocal images were acquired using a Leica TCS-SP8 confocal microscope equipped with LAS-X software (v4.13). Maximum intensity z-projections were performed using ImageJ software (1.53t). Imaging conditions were kept consistent between animals/treatment groups.

For immunohistochemical staining, endogenous peroxidase activity in free-floating sections was quenched in 3% hydrogen peroxide for 7 min. Sections were incubated in 10% normal serum or Mouse on Mouse blocking solution for 1 h, probed with primary antibody against Iba1 (WAKO, 019-19741, 1:500) overnight at room temperature and then incubated with biotinylated secondary antibody for 1 h. Peroxidase-based staining with the chromogen 3,3′-diaminobenzidine (DAB) was performed according to the manufacturer’s instructions (Vector Laboratories). Immunohistochemical staining for Iba1 was performed at the same time under identical staining conditions in each animal/treatment group, thereby eliminating any potential variability due to the histology process. Stained brain slices were mounted on slides, cleared in xylene and coverslipped in DPX mounting media. Images were acquired using a Zeiss Axioskop 2 light microscope (Zeiss, Thornwood, NY, USA) with the Stereo Investigator Version 2017 software. Imaging conditions were kept consistent between animals/treatment groups.

### 2.5. Tissue Homogenization and Protein Extraction

Brain tissues were homogenized by sonication in 200 μL of ice-cold PBS containing protease inhibitors (EDTA-free complete protease inhibitor cocktail, Roche). The samples were clarified by centrifugation at 15,000× *g* for 10 min at 4 °C and the PBS-soluble supernatant was collected. Pellets were resuspended in 200 μL of PBSTr (1X PBS + 1% Triton X-100 + 5 mM EDTA + 1:100 protease/phosphatase inhibitor cocktail), followed by sonication and centrifugation. After collecting the clarified PBSTr-soluble supernatant, the pellet was resuspended in SDS (1X PBS + 2% SDS + 5 mM EDTA + protease/phosphatase inhibitor cocktail), centrifuged once more and to obtain the SDS-soluble fraction. Pooled sample aliquots were prepared by mixing the PBS and PBSTr fractions at a ratio of 1:1 for protein analysis. All samples were stored at −30 °C.

### 2.6. Western Blotting

Sample protein concentrations were quantified by BCA assay using a BSA standard curve. Protein concentrations were equalized in PBS to between 5–30 μg protein load per well. Proteins were denatured in reducing sample buffer and samples were heated at 70 °C for 5 min. Proteins were separated by SDS-PAGE using precast Criterion TGX 4–20% polyacrylamide stain-free gels containing a trihalo compound for stain-free protein detection by UV-fluorescence (Bio-Rad, Hercules, CA, USA). Electrophoresis was performed at 75 V for 10 min and then 150 V for 1 h. Separated proteins were transferred to PVDF membranes by semi-dry transfer at 2.5 A for 7 min. Membranes were blocked in 5% nonfat dry milk in Tris-buffered saline with 0.1% Tween 20 (TBS-T) for 30 min at room temperature and were probed overnight at 4 °C with primary antibodies against the following target proteins: ABCA1 (Abcam, ab7360, 1:1000), ApoE (Abcam, ab183597, 1:1000), C1q (Abcam, ab182451, 1:500), GFAP (EMD Millipore, MAB360, 1:2000), Iba1 (Abcam, ab178846, 1:1000), LAMP1 (Abcam, ab208943, 1:1000), LC3 (Millipore, ABC232, 1:1,000), LRP1 (Abcam, ab92544, 1:1000), NPC1 (Abcam, ab134113, 1:2000), p62 (Cell Signaling Technologies, 5114S, 1:1000). The membranes were washed five times in TBS-T for 5 min each and incubated with HRP-conjugated secondary antibodies for 1 h at room temperature. Membranes were washed five times in TBS-T for 5 min each and incubated with ECL for the digital detection of immunoreactive bands. Band intensities were quantified using Image Lab Software version 6.1 (BioRad) and normalized to a representative set of bands detected by total protein UV-fluorescence (shown in Western blot figures), reported as values relative to the WT vehicle control group.

### 2.7. Statistical Analysis

Statistical analyses were performed in GraphPad Prism software version 9.4.1. All data are expressed as mean ± SEM. One-way ANOVAs followed by Tukey’s post hoc analysis of statistically significant distributions were used. *p* values < 0.05 were considered significant for all analyses.

## 3. Results

### 3.1. Prolonged Glycolipid Elevation by GBA1 Inhibition Increases Brain ApoE Protein

**Figure 1:**
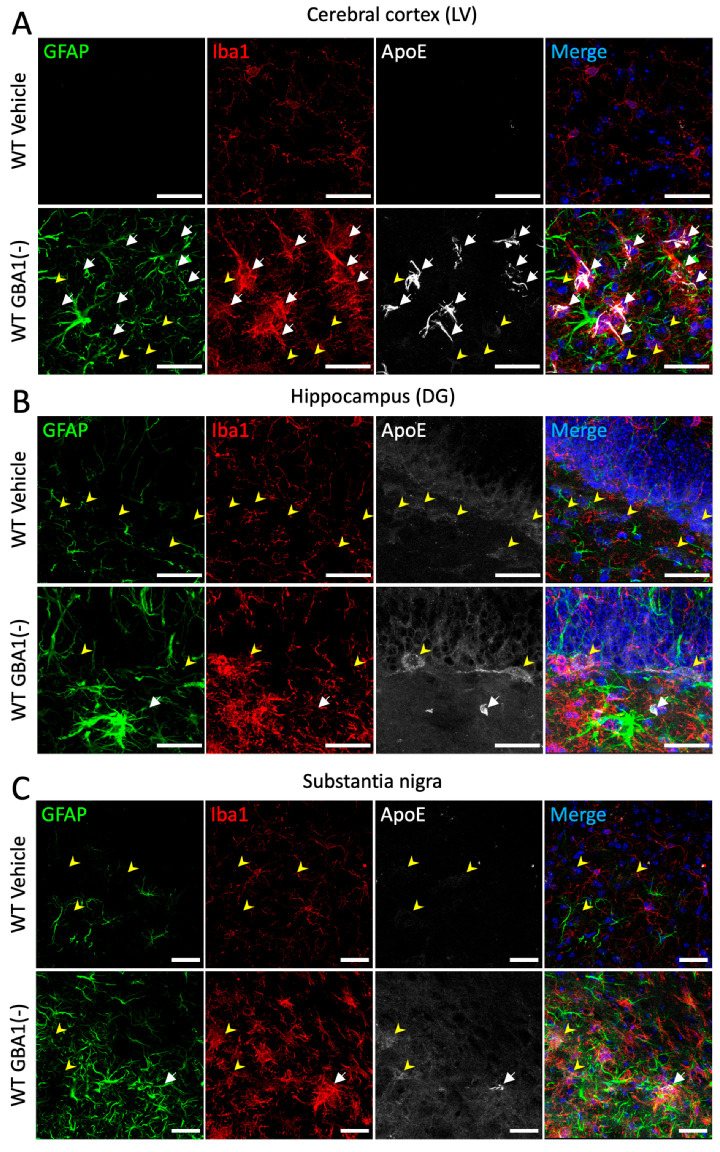
Prolonged glycolipid elevation by GBA1 inhibition increases ApoE protein in cortical layer V, hippocampus and substantia nigra. Brain slices from 3 mo WT mice after daily i.p. GBA1 inhibitor, CBE or vehicle for 18 days were stained for ApoE and co-stained with astrocyte marker GFAP and microglial marker Iba1. Images showing cortical layer V (LV) (**A**), the hippocampus dentate gyrus (DG) (**B**) and the substantia nigra (**C**) are presented. Yellow arrowheads annotate cells with punctate ApoE and white arrows show strong ApoE labeling. Nuclear staining by Hoechst is only shown in merged images. Scale bar = 50 μm. GBA1(-), GBA1 inhibition.

Brain cellular lipid transport and metabolic pathways are activated in response to increased lipid levels. Given the critical function of ApoE to transport lipids between brain cell types, we predicted that sustained brain glycolipid elevations caused by GBA1 inhibition would cause brain ApoE responses. To investigate such brain ApoE responses to glycolipid elevations caused by GBA1 inhibition in vivo, WT mice received daily i.p. injections of an irreversible and brain-permeable GBA1 inhibitor (conduritol β epoxide, CBE) or vehicle for 18 days. At post mortem, to assess whether brain ApoE levels are altered in WT mice following GBA1 inhibition, brain sections were stained for ApoE and co-stained with astrocyte marker GFAP and microglial marker Iba1 (Figure 1). Markedly increased ApoE labeling was particularly seen in cortical layer V (Figure 1A), and there were also increases in the hippocampus (Figure 1B) and substantia nigra (Figure 1C) after GBA1 inhibition (GBA1(-)) compared to control mice that received the vehicle. Further analysis revealed that the majority of ApoE colocalized with microglia in cortical layer V, whereas ApoE did not colocalize with astrocytes. Increased punctate ApoE labeling, which did not overlap with Iba1, was detected in each brain region studied, especially in the dentate gyrus. Morphologically, microglia and astrocytes had markedly larger sizes reflecting their biological activation after GBA1 inhibition in cortical layer V, the hippocampus and substantia nigra in the brains of WT mice. These results revealed that, following sustained systemic GBA1 inhibition, there were increased ApoE levels in several brain regions that were concomitant with microglial and astrocyte activations in these regions. Given these findings of co-dependency between GBA1 and ApoE, we decided to investigate this relationship further.

### 3.2. Loss of ApoE Amplified Brain Regional Microgliosis in Response to GBA1 Inhibition

**Figure 2:**
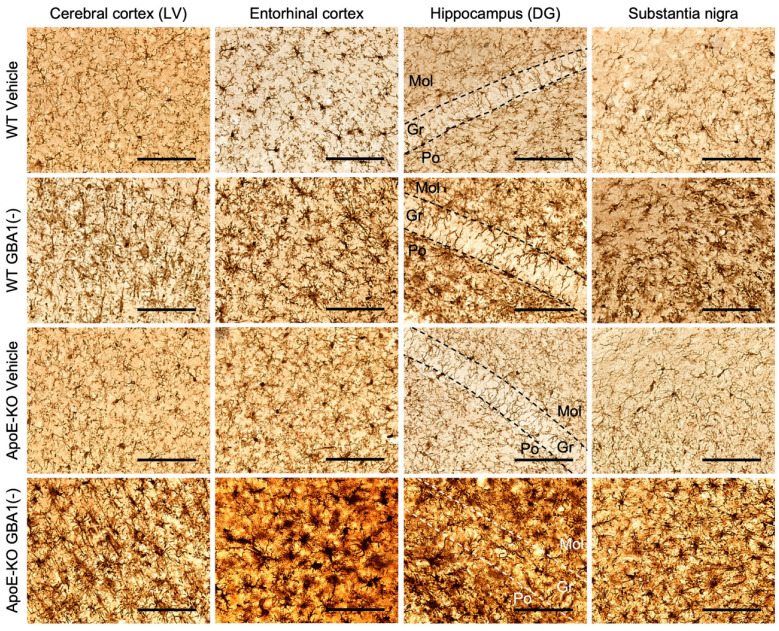
**Loss of ApoE amplified microgliosis in response to GBA1 inhibition.** Representative Iba1 DAB stains are shown of brain slices from 3 mo WT and ApoE-KO mice that received daily i.p. GBA1 inhibitor, CBE or vehicle for 18 days. Scale bar = 100 μm. LV, layer V; DG, dentate gyrus; Mol, molecular layer; Gr, granule cell layer; Po, polymorph layer; GBA1(-), GBA1 inhibition.

Based on the known functions of ApoE in intercellular lipid transport, we hypothesized that following GBA1 inhibition with elevated glycolipids, compensatory functional interactions between ApoE and other lipid regulatory systems could occur. Lipid elevations in the brain are also associated with innate and other inflammatory responses [4,33]. To test if the absence of ApoE exacerbated brain inflammation and glial activation caused by sustained GBA1 inhibition, WT and ApoE-KO mice were dosed with daily i.p. CBE or vehicle for 18 d. Confirmation of GBA1 inhibition in the brain of WT and ApoE-KO was determined by measuring lysosomal GBA1 enzymatic activity in cerebral cortex lysates, and showed that GBA1 activity was reduced by >75% in mice treated with CBE/GBA1 inhibition (WT mice with GBA1 inhibition = 15.75% of WT vehicle-treated mice (*p* < 0.05); ApoE-KO vehicle-treated mice = 107.15% of WT vehicle-treated animals (*p* > 0.05); ApoE-KO mice with GBA1 inhibition = 25.20% (*p* < 0.05) of WT vehicle-treated animals). Brain slices were stained for Iba1 (Figure 2) and histological examination of Iba1-labeled microglia showed morphological changes consistent with the robust activation of microglia across multiple brain regions. Activated microglia showed sub-regional organization and were particularly prominent in the cerebral cortex, hippocampus and substantia nigra, as previously observed [21]. In the cerebral cortex, the greatest levels of microglial activation were seen within layer V. Activated microglia after GBA1 inhibition were hypertrophic, with long processes in parallel organization. This microglial activation and organization were not observed in the brains of mice that received the vehicle. In addition, microglial activation was prominent in the molecular and polymorph layers of the hippocampus dentate gyrus and the substantia nigra. In each brain region observed, microglial activation was greater in ApoE-KO mice after GBA1 inhibition relative to WT. The pattern of microglial activation within brain sub-regions was similar between WT and ApoE-KO mice. These data indicate that the overall inflammatory response to sustained glycolipid elevations is amplified in the absence of ApoE.

### 3.3. Loss of ApoE Elevated Brain Regional C1q Deposition after GBA1 Inhibition

**Figure 3:**
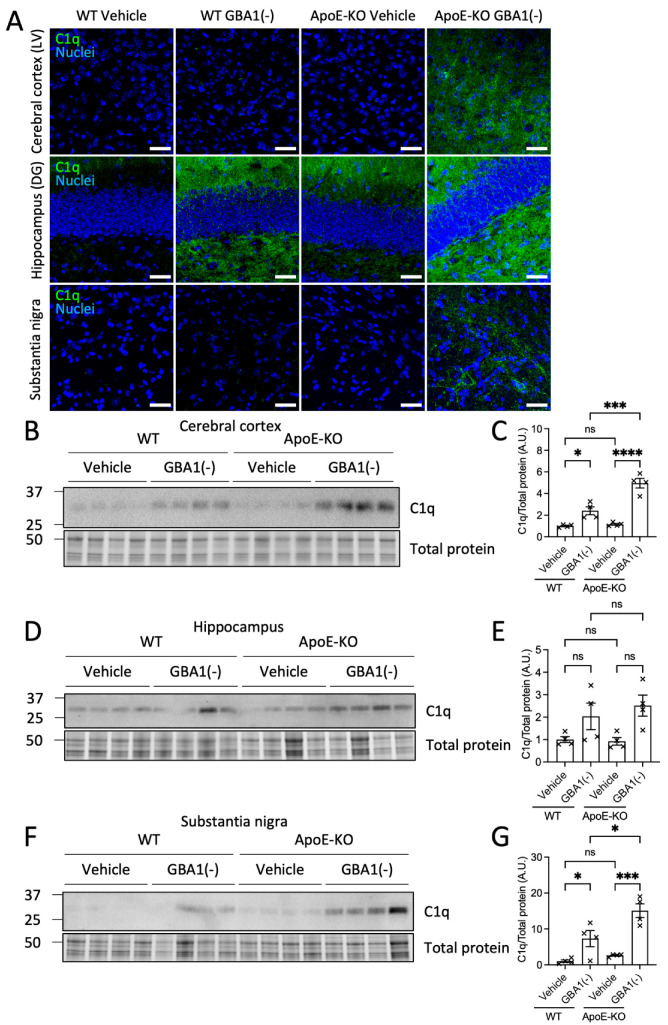
GBA1 inhibition increased C1q deposition in the cerebral cortex, the hippocampus and the substantia nigra, and was amplified by loss of ApoE. (**A**) Representative images of sections from 3 mo WT and ApoE-KO mice after daily i.p. GBA1 inhibitor, CBE or vehicle for 18 days stained for C1q. For presentation, nuclear staining by Hoechst is only shown in merged images. LV, layer V; DG, dentate gyrus. Scale bar = 50 μm. (**B**–**G**) Brain regions were dissected and homogenized for analysis by Western blot. Four animals from each group were analyzed. Total protein was detected in-gel by stain-free UV-fluorescence to visualize protein loading. Uncropped blots can be found in Supplementary Materials. Histograms represent mean ± SEM. ns, not significant; *, *p* < 0.05; ***, *p* < 0.001; ****, *p* < 0.0001. GBA1(-), GBA1 inhibition.

To examine acute and sustained innate inflammatory responses in this study, mouse brain slices were stained for the complement protein C1q, a marker of the inflammatory complement response (Figure 3A). C1q immunolabeling was nearly undetectable in the brains of WT and ApoE-KO mice that received the vehicle. GBA1 inhibition caused increased C1q labeling in the hippocampus dentate gyrus of WT mice, whereas no difference in C1q labeling was detected in cortical layer V or in the substantia nigra. By contrast, GBA1 inhibition increased C1q labeling in cortical layer V, the hippocampus dentate gyrus and the substantia nigra of ApoE-KO mice (Figure 3A). Cell membrane labeling by C1q was observed in cortical layer V and in the substantia nigra of ApoE-KO mice after GBA1 inhibition, which may represent C1q deposits on the membranes of cells destined for degeneration by immune mechanisms, as previously observed [21]. Next, brain regional levels of C1q protein were quantified by Western blot (Figure 3B–G). Quantification of C1q protein levels revealed significantly increased C1q levels after GBA1 inhibition in the cerebral cortex and substantia nigra that were amplified in the absence of ApoE. Similar trends in C1q responses were also seen in the hippocampus. The amplified C1q elevations after GBA1 inhibition in brains without ApoE demonstrate that the brain inflammatory complement response to prolonged glycolipid stress is increased in the absence of ApoE.

### 3.4. Loss of ApoE Increased LAMP1 Lysosomal Labeling Associated with Microglia after GBA1 Inhibition

**Figure 4:**
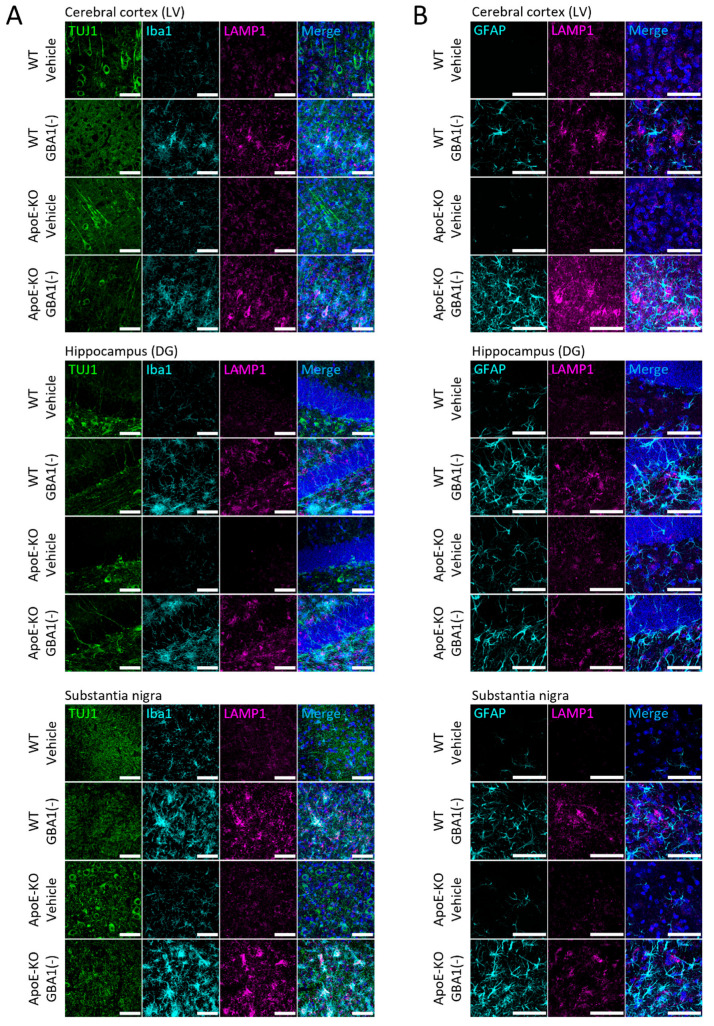
Loss of ApoE increased GBA1 inhibition-induced LAMP1 labeling in microglia within cortical layer V, the hippocampus dentate gyrus and substantia nigra. Representative images of sections from 3 mo WT and ApoE-KO mice after daily i.p. GBA1 inhibitor, CBE or vehicle for 18 days stained for TUJ1, Iba1 and LAMP1 (**A**), or GFAP and LAMP1 (**B**). Images of cortical layer V (LV), the hippocampus dentate gyrus (DG) and substantia nigra are shown. Nuclear staining by Hoechst is only shown in merged images. Scale bars = 50 μm. GBA1(-), GBA1 inhibition.

Previous data have shown that GBA1 inhibition by CBE causes glycolipid substrates to accumulate in the brain [21,34,35,36]. As glycolipid substrates are stored when lysosomal GBA1 is inhibited, we predicted that CBE would cause the number of lysosomes to increase. To test this, lysosome membranes were stained for LAMP1 (Figure 4A). LAMP1 levels and distribution were similar between WT and ApoE-KO mice at baseline and mainly localized to neuron cell bodies. GBA1 inhibition increased LAMP1 protein immunolabeling that coincided with Iba1 in multiple brain regions, including cortical layer V, the hippocampus dentate gyrus and substantia nigra in WT and ApoE-KO mice. Importantly, LAMP1 labeling in activated microglia increased to a larger degree in ApoE-KO mice after GBA1 inhibition, compared to WT mice (Figure 4A). The increased lysosomal responses concomitant with greater microglial activation in the absence of ApoE following GBA1 inhibition supports the hypothesis of increased functional demand for brain cellular lipid transport by ApoE following GBA1 inhibition.

Levels of astrocyte activation were investigated by staining for GFAP (Figure 4B). Morphologically, astrocyte activation caused by GBA1 inhibition was amplified by the loss of ApoE in cortical layer V and in the substantia nigra. Differences in the LAMP1 labeling of lysosomes in astrocytes after GBA1 inhibition and in the absence of ApoE were assessed by staining with LAMP1 and GFAP. Increases in LAMP1 labeling were seen after GBA1 inhibition, but did not occur within astrocytes in WT and ApoE-KO mice (Figure 4B). The elevated LAMP1 observed after GBA1 inhibition in ApoE-KO mice may be a functional marker of microglial responses to GBA1 inhibition, which are amplified when ApoE is absent.

### 3.5. Increased Lysosome, but Not Autophagosome, Membrane Formation after GBA1 Inhibition Was Amplified by Loss of ApoE

**Figure 5:**
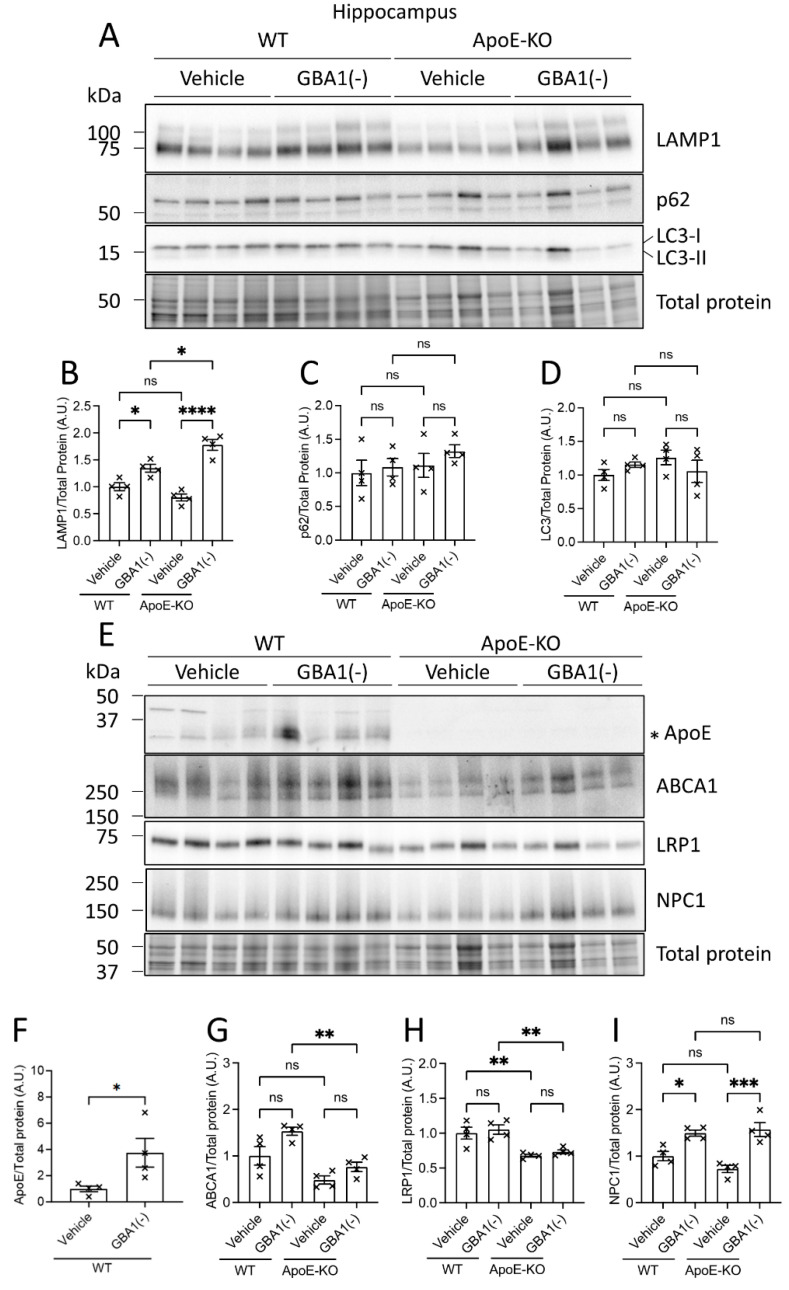
Endolysosome membrane formation and NPC1 protein levels increased after GBA1 inhibition, and ABCA1 and LRP1 protein levels were reduced in the absence of ApoE. (**A**–**I**) WT and ApoE-KO mice were injected daily for 18 days with 100 mg/kg i.p. GBA1 inhibitor, CBE. Hippocampi were isolated and homogenized for analysis by Western blot. LC3-I and LC3-II bands were quantified together to measure total LC3 levels (**D**). The band quantified for ApoE is shown by an asterisk (**E**). Four animals from each group were analyzed. Total protein was detected in-gel by stain-free UV-fluorescence to visualize protein loading. Uncropped blots can be found in Supplementary Materials. Histograms represent mean ± SEM. ns, not significant; *, *p* < 0.05; **, *p <* 0.01; ***, *p <* 0.001; ****, *p* < 0.0001. GBA1(-), GBA1 inhibition.

The increased LAMP1 labeling observed within activated microglia after GBA1 inhibition indicates an increased demand for lysosomal functions that may be impinged by glycolipid substrate accumulation. The degradation of cellular proteins and organelles such as mitochondria by autophagy is dependent on lysosomal function [37]. Protein markers of lysosome and autophagosome membranes were quantified to investigate the impact of sustained GBA1 inhibition on autophagy. While in the histological analysis we observed consistent and parallel changes in LAMP1 labeling in the cerebral cortex, hippocampus and substantia nigra following GBA1 inhibition, the mechanistic biochemical analyses were performed in the hippocampus, as this is a major region of interest in LBD and related disorders. Whilst the histological analysis informed discrete anatomical localization of protein labeling in WT and ApoE-KO mice in response to GBA1 inhibition, parallel biochemical analyses provided increased sensitivity and quantitation of protein levels. Consistent with the increased LAMP1 labeling in the brain caused by GBA1 inhibition (see Figure 4), LAMP1 protein levels were increased after GBA1 inhibition, and this increase was elevated further in the hippocampus of ApoE-KO mice (Figure 5A,B). By contrast, GBA1 inhibition did not cause changes in p62 or LC3 protein levels in either WT or ApoE-KO mice (Figure 5A,C,D). This indicates that while lysosomal membranes were increased in response to GBA1 inhibition, there were no observable changes in autophagy given that protein levels of p62 and LC3 (I and II) were unchanged.

### 3.6. ABCA1 and LRP1 Protein Levels Were Reduced in the Absence of ApoE, Whereas NPC1 Protein Levels Increased in Response to GBA1 Inhibition

The observed increases in brain ApoE levels caused by GBA1 inhibition indicate that ApoE function is potentially important to mitigate excessive cellular lipid storage. Mechanisms for cellular lipid transfer by ApoE involve integrated cell secretory and endocytic networks. Briefly, ApoE binds lipids that are exported by cell surface lipid transporters, such as ABCA1 [38], and endocytosis facilitated by lipoprotein receptors, such as LRP1 [39]. Internalized lipid cargo is then metabolized by lysosomal lipid hydrolases and exported by lysosomal lipid transporters, such as NPC1 [40]. The levels of these lipid transport and receptor proteins were biochemically quantified to determine how GBA1 inhibition influences lipid transport mechanisms that depend on ApoE (Figure 5E–I). Baseline levels of ABCA1 and LRP1 proteins were significantly lower in the hippocampus of ApoE-KO mice (Figure 5E,G,H), suggesting that cellular lipid exchange is reduced in the absence of ApoE. There were trends in increased ABCA1 levels after GBA1 inhibition in the hippocampus of WT and ApoE-KO mice. Then, to obtain an indication of intracellular lipid transport at the level of the lysosome, levels of the cholesterol and putative sphingolipid transporter NPC1 were quantified (Figure 5E,I). NPC1 protein levels were significantly elevated after GBA1 inhibition, and this increase was similar between WT and ApoE-KO mice. No difference in brain NPC1 protein levels were observed between WT and ApoE-KO, in contrast to the reduced ABCA1 and LRP1 levels in the brains of ApoE-KO mice at baseline. The findings that ABCA1 and LRP1 levels decreased in the absence of ApoE demonstrate that levels of brain lipid transporters and apolipoprotein receptors that function to transfer lipids between cells are decreased. By contrast, levels of the lysosomal cholesterol transporter NPC1 increased following GBA1 inhibition and was not changed by the absence of ApoE, indicating that lysosomal cholesterol transport responses triggered by GBA1 inhibition were unaffected by the absence of ApoE.

### 3.7. ApoE and Complement Factor C1q Protein Levels Were Elevated in the Brains of NPC1^−/−^ Mice

**Figure 6:**
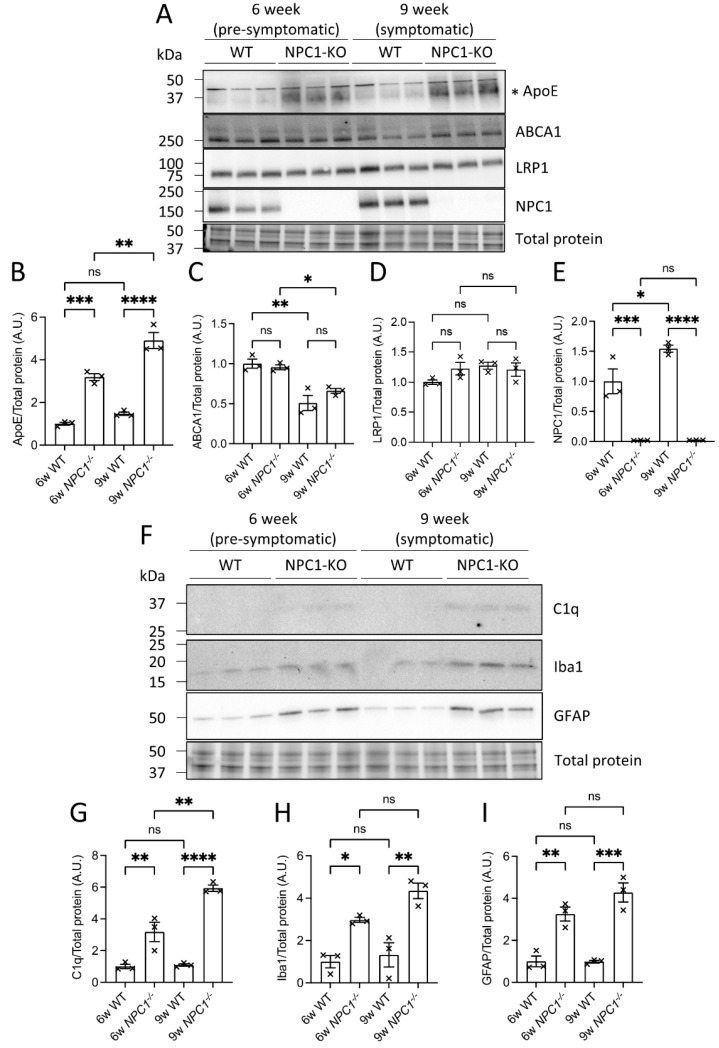
**ApoE and complement factor C1q protein levels were elevated in mouse brains in the absence of NPC1.** (**A**–**I**) Brains from WT and *NPC1^−/−^* mice were extracted and one hemisphere was homogenized for analysis by Western blot. The band quantified for ApoE is shown by an asterisk (**A**). Three animals from each group were analyzed. Total protein was detected in-gel by stain-free UV-fluorescence to visualize protein loading. Uncropped blots can be found in Supplementary Materials. Histograms represent mean ± SEM. ns, not significant; *, *p* < 0.05; **, *p* < 0.01; ***, *p* < 0.001; ****, *p* < 0.0001.

The observed elevations of brain lipid transporters ApoE and NPC1 after GBA1 inhibition in these experiments are evidence of an increased demand to transport lipids during lysosomal lipid storage. We and others have previously shown that regionally elevated brain and cellular glycolipids and triglycerides cause inflammatory responses that are linked to neurodegenerative events [17,41]. We hypothesized that ApoE and NPC1 are functionally linked in the removal of cellular lipids to prevent inflammation and damage caused by excess lipid storage. To examine the link between ApoE and NPC1, brain ApoE and inflammatory C1q protein levels were measured in the context of lysosomal cholesterol storage caused by NPC1 deficiency using mice with genetic disruption of the *NPC1* gene (*NPC1^−/−^*) [42]. The NPC1 protein was completely undetectable in the brains of *NPC1^−/−^* mice (Figure 6A,E). ApoE protein levels were increased in the brains of pre-symptomatic *NPC1^−/−^* mice aged 6 weeks and increased further at 9 weeks of age (Figure 6A,B). These increases in brain ApoE levels were not accompanied by changes in ABCA1 or LRP1 (Figure 6A,C,D), suggesting that ApoE drives the export of lipids from cells when NPC1 is absent. Complement C1q and markers of microglial and astrocyte activation Iba1 and GFAP, respectively, were quantified since increased lipid load is associated with complement activation and inflammation. The analysis showed that C1q, Iba1 and GFAP levels were increased at 6 weeks and increased further at 9 weeks in *NPC1^−/−^* mice (Figure 6F–I), consistent with previous reports in mice with NPC1 loss of function [43].

## 4. Discussion

### 4.1. Summary of Experiments and Findings

These in vivo experiments tested the functional brain responses to GBA1 inhibition and loss of ApoE in mice models. Glycolipid stress caused by GBA1 inhibition induced ApoE expression in several brain regions of WT mice, including the cerebral cortex, hippocampus and substantia nigra. When this glycolipid stress was applied in mice with the deletion of ApoE, it exacerbated microgliosis and to some extent astrocytosis in the brain regions examined. Notably, in mice lacking ApoE, marked elevations of C1q protein were observed in the same brain regions that were sensitive to GBA1 inhibition. Brain regional baseline levels of the lipid transporter ABCA1 and the ApoE receptor LRP1 were reduced in the absence of ApoE, and following GBA1 inhibition, NPC1 levels were increased. To understand the connection between intracellular lipid transport and extracellular lipid exchange by ApoE, the intracellular lysosomal lipid transporter NPC1 was investigated in mice. Interestingly, the absence of NPC1 in mice caused elevations in brain ApoE and C1q. These data show that ApoE and C1q protein are recruited in response to both GBA1 inhibition and NPC1 inhibition, illustrating a conserved, adaptive and integrated cellular response to increased lipid loads.

### 4.2. Brain ApoE Lipid Transport Function Is Critical to Adapt to Lipid Perturbations

A central finding of these experiments is that sustained GBA1 inhibition in the mouse brain triggered increases in ApoE levels in the context of microglial and astrocyte activation. The increases in ApoE levels and glial activation were observed in multiple brain regions relevant to dementia syndromes, including layer V of the cerebral cortex, the dentate gyrus of the hippocampus and the substantia nigra. The results shown are critical to the understanding of the physiological role of ApoE because they clearly demonstrate that cellular increases in ApoE levels and function are induced by glycolipid stress. Such increases in ApoE levels and function are likely triggered to adapt to cellular lipid perturbations that occur during experimental GBA1 inhibition and the age-dependent decline in brain lysosomal lipid hydrolase activities [10,18,19,21,44]. Based on these new data, we believe that cellular adaptive lipid transport mechanisms, such as ApoE, are engaged to respond to upstream cellular perturbations, such as lipid elevations, inflammation, mitochondrial dysfunction or oxidative stress [4,10,41,45].

Glycolipid elevations that occur in the aging brain and in LBD and related dementias impact several nodes of cell biology, including membrane formation, membrane dynamics, lipid trafficking, organelle functions and inflammation [10,18,21,41,45]. Previous findings have shown that GBA1 pharmacological inhibition by CBE in mice results in a complete and sustained reduction in GBA1 activity in the brain, with concomitant elevations in glycolipids and neuroinflammatory responses, in multiple brain regions [21,22,46]. The significant finding in the current study that the absence of brain ApoE amplified the inflammatory consequences of brain glycolipid elevations highlights important physiological interactions between lipid transport and inflammation. Lipid transport between neurons and glia in the brain is essential to prevent excessive intracellular lipid accumulation, remove toxic lipid products, such as peroxidated lipids, and ensure that the cellular demand for lipid precursors is met [4,9,24,41]. Previous studies have shown that GBA1 inhibition in the mouse brain recapitulates the redistribution of neutral lipids between dopaminergic neurons and glia in the substantia nigra of human PD patients, with increased neutral lipid content in neurons and microglia and decreased neutral lipids in astrocytes [18]. In the current study, the new finding that brain ApoE levels are increased following GBA1 inhibition could indicate that neutral lipid redistribution between neurons and glia is facilitated by ApoE in the brains of PD patients and in mouse models of GBA1 inhibition.

### 4.3. Do ApoE Responses to Glycolipid Stress Limit Complement Factor C1q Activation?

Another major result of the experimental paradigms reported here is that sustained GBA1 inhibition in the mouse brain triggers increases in levels of inflammatory complement factor C1q, in parallel with significant increases in ApoE levels and glial activation. Importantly, the absence of ApoE amplified brain inflammatory C1q levels and the activation of microglia and astrocytes following GBA1 inhibition. The observed elevation in C1q in the brain is a marker of an inflammatory milieu that can cause damage to and potentially eliminate synapses. Classical complement factors including C1q are required in brain development for synapse pruning by microglia to refine neuronal circuitry [47]. Interestingly, C1q levels increase during aging in mouse and human brains, particularly in the hippocampus, and C1q contributes to age-associated cognitive decline in mice [48]. Increased C1q levels in the hippocampus of AD brains and experimental models are associated with increased synapse elimination by microglial phagocytosis [49,50]. In the current study, the discovery that brain C1q elevations were amplified by the absence of ApoE revealed that ApoE performs critical protective functions to quench C1q during glycolipid stress. This observation is supported by recent data that complement factor C1q binds to lipidated ApoE and that several complement factors, including C1q, C3 and C5, are associated with lipid deposits that form in the brain during conditions of high lipid load and ApoE insufficiency [17].

### 4.4. ApoE Function Is Critical following Astrocyte and Microglial Activation

Sustained GBA1 inhibition in the mouse brain caused the activation of astrocytes and microglia across several disease-relevant brain regions, including the cortex, hippocampus and substantia nigra. The levels of glial activation, which correlate with increased inflammatory output, were amplified in the absence of ApoE, highlighting that lipid transport by ApoE is closely linked to the regulation of inflammation in the brain. Abnormal levels and perturbed glycolipid and neutral lipid regulation within neurons susceptible to degeneration in LBD and related dementias likely signal to surrounding cells, including microglia and astrocytes [21,41]. Astrocytes are the main source of brain ApoE during homeostasis, which functions to maintain lipid balance between neurons and astrocytes and eliminate oxidized lipids [5,9,12]. Increased microglial ApoE production occurs during inflammation and injury [15,51,52]. Interestingly, it has been shown that the pharmacological depletion of microglia in the mouse brain caused compensatory increases in hippocampus ApoE levels in astrocytes and even neurons under extreme proteotoxic stress [53]. In the context of the present findings, the increased levels of microglial ApoE observed during glycolipid stress is consistent with increased ApoE that is typically seen in activated microglia triggered by inflammation.

The current findings showed increased levels of the endolysosomal membrane marker LAMP1 within activated microglia, indicating that the demand for lysosomal function is increased in microglia during glycolipid stress and is amplified in the absence of ApoE. Microglia are the brain resident immune cells that have functional similarities with macrophages. Cells that internalize large volumes of content, either in bulk by phagocytosis or in pieces through high rates of endocytosis or related mechanisms, have an innate high demand for endolysosomal function. Internalized cargo is trafficked from endosomes to lysosomes for degradation. For phagocytic cells, this mechanism is important to survey the local environment for signs of damage or infection to swiftly activate appropriate immune response mechanisms [54]. The high lysosomal function of microglia also makes microglia ideal recipients for ApoE lipid particles for the efficient turnover of lipids when lipids are elevated.

### 4.5. Lipid Transport and Lysosomal Mechanisms Integrate Intracellular and Extracellular Lipid Adaptations

ApoE is a secreted lipoprotein that binds lipids that are exported by lipid transporter proteins that reside in cell membranes. ABCA1 is the main lipid transporter that transports cholesterol to ApoE, in addition to lipid transporters ABCA7 and ABCG1 that also transport cholesterol and phospholipids, which may also be required for the lipidation of ApoE [23,55]. Lipidated ApoE particles in the extracellular space are bound by cell surface lipoprotein receptors, such as LRP1, LRP8 and TREM2 [15], which facilitate the internalization of lipoprotein particles into cells. Protein levels of the lipid transporter ABCA1 and the ApoE receptor LRP1 were reduced in the brains of unchallenged ApoE-KO mice, indicating that extracellular lipid exchange in the brain is significantly reduced in the absence of ApoE under homeostatic conditions. These deficits in extracellular lipid transport caused by ApoE insufficiency could reduce the cellular capacity to adapt to lipid elevations.

Captured ApoE-lipid particles are trafficked through the endosomal pathway for digestion by lysosomal hydrolases. Cholesterol is a major lipid that is transported by ApoE and is exported from lysosomes by the cholesterol transporter NPC1 through a mechanism that may involve the transfer of cholesterol between membranes at contact sites between organelles [42]. In the cell biology of lipid metabolism and distribution, the lysosomes play a critical role in glycolipid and complex glycolipid breakdown and homeostasis [41,56]. Clinically, our previous studies have demonstrated that lysosomal lipid hydrolase activities in the brain, including GBA1, are reduced in human and animal aging, and further reductions in hydrolase activities in PD lead to glycolipid substrate accumulation [19,20,44]. Interestingly, ApoE levels increased during primary glycolipid elevations caused by GBA1 inhibition. It is likely that lipids including cholesterol, triglycerides, and glycolipids in the membrane or other vesicular compartments are regulated in concert. During metabolic and lipid disturbances, the inflammatory responses seen in glial cells indicate that the system is responding to signals that are outside the normal physiological range. Given the present findings that ApoE is linked to glycolipid changes due to GBA1 inhibition, the overall regulation of cholesterol and transport and glycolipid levels indicate such important functional relationships.

When lipid degradation fails and lipids accumulate, unresolvable lipid formations may attract intrinsically hydrophobic and lipid-binding proteins that are associated with neurodegenerative diseases. We previously observed that insoluble and proteinase-resistant α-synuclein inclusions form in the substantia nigra when brain GBA1 inhibition in mice is sustained for 28 d [21]. Experimental GBA1 gene therapy in an α-synucleinopathy mouse model increased brain GBA1 activity and prevented α-synuclein aggregation [32,57,58,59,60]. In addition, increasing β-hexosaminidase activity in *HEXB*-deficient Sandhoff mouse models reduced the association of α-synuclein with lipids [61]. These previous findings demonstrate that lipid-associated α-synuclein formations are increased when either lipid or α-synuclein levels are increased, and may be reversible if lipid levels are reduced. Lipid binding by α-synuclein can be attributed to its apolipoprotein-like domain [4], and could function to bind lipids during dynamic changes in cellular lipid levels. ApoE is the primary apolipoprotein expressed by the brain, which could mean that ApoE also binds lipid aggregates that cannot be resolved due to deficits in GBA1 or other lysosomal enzyme functions.

## 5. Conclusions

ApoE function is widely studied in blood, liver and peripheral systems related to cardiovascular disease [62,63]. The pathophysiological role of ApoE in cholesterol lipid function in the brain is relatively less well understood. However, the remarkable difference in late-onset Alzheimer’s disease risk for carriers of ApoE2/3 variants versus the increased risk of ApoE4 variant carriers [1,2,3,63,64,65] highlights a critical need for understanding of the specific and general roles of ApoE in brain cell biology and brain tissue cell pathophysiology. Recently, a genetic and post-mortem study revealed that, at the cellular level in an ApoE3 variant human carrier brain, high ApoE gene expression overlapped with less regional brain tau pathology in autosomal dominant Alzheimer’s disease [3]. ApoE and GBA1 are the most significant combined genetic risk factors for LBD and related dementias [1,2], and the in vivo experiments reported here tested functional and brain regional responses to GBA1 inhibition and loss of ApoE in mice models. The new findings are relevant to understanding the integration of ApoE function with brain cell responses to varied glycolipid and cholesterol stresses. In particular, brain ApoE levels were increased by glycolipid stress after GBA1 inhibition and by cholesterol storage caused by NPC1 deficiency, showing that brain ApoE, GBA1 and NPC1 lipid biological functions are interconnected in vivo. The absence of ApoE amplified brain regional glial and inflammatory responses to glycolipid stress caused by GBA1 inhibition, showing that adaptive increases in ApoE function to transport lipids are important to mitigate downstream cellular reactions. These current findings indicate that the removal or reduction of ApoE would likely be detrimental to brain function. Indeed, baseline levels of the cell surface lipid transporter ABCA1 and ApoE receptor LRP1 levels were reduced in the absence of ApoE, indicating reduced brain cellular lipid exchange in the absence of ApoE. Further studies are required to investigate how adaptive lipid mechanisms during glycolipid stress could become maladaptive in aging and lead to neurodegeneration.

These new insights into brain ApoE function during glycolipid stress reported here emphasize the importance of adaptive and interconnected cellular lipid metabolic and transport mechanisms, which are activated by lipid stresses, to redistribute lipids and mitigate downstream inflammatory activations in the brain. Reduced cellular lipid exchange caused by deficits in ApoE function amplified cellular and inflammatory consequences of lipid stress, and emphasized ApoE as a therapeutic target in age-related neurodegeneration such as LBD and other dementia syndromes.

## Data Availability

The data generated during the current study are available from the corresponding author(s) on reasonable request.

## Supplementary Materials

The following supporting information can be downloaded at https://www.mdpi.com/article/10.3390/cells12212564/s1, Figures S1–S21: Uncropped images for western blots used to create representative gel images in Figure 3, Figure 5 and Figure 6.

## Abbreviations

ABC, adenosine triphosphate (ATP)-binding cassette; ANOVA, analysis of variance; ApoE, Apolipoprotein E; ApoE-KO, Apolipoprotein E-knockout; BCA, bicinchoninic acid; BSA, bovine serum albumin; CBE, conduritol-β-epoxide; DAB, 3,3′-diaminobenzidine; DG, dentate gyrus; DMSO, dimethyl sulfoxide; DPX, dibutylphthalate polystyrene xylene; ECL, enhanced chemiluminescence; EDTA, ethylenediamine tetraacetic acid; GBA1, β-glucocerebrosidase; GBA1(-), β-glucocerebrosidase inhibition; GFAP, glial fibrillary acidic protein; Gr, granule cell layer; HEXB, hexosaminidase β-subunit; HRP, horseradish peroxidase; i.p., intraperitoneal; IACUC, Institutional Animal Care and Use Committee; Iba1, ionized calcium-binding adapter molecule 1; LAMP1, lysosomal-associated membrane protein 1; LBD, Lewy body dementia; LC3, microtubule-associated protein 1A/1B-light chain 3; LRP, low density lipoprotein receptor (LDLR)-related protein; LV, layer V; Mol, molecular layer; n.s., not significant; NPC1, Niemann-Pick disease, type C1; PBS, phosphate-buffered saline; PBSTr, phosphate-buffered saline Triton X-100; PD, Parkinson’s disease; Po, polymorph layer; PVDF, polyvinylidene fluoride; SDS, sodium dodecyl sulfate; SDS-PAGE, sodium dodecyl sulfate polyacrylamide gel electrophoresis; SEM, standard error of mean; TBS-T, tris-buffered saline Tween 20; TREM2, triggering receptor expressed on myeloid cells 2; TUJ1, β-III tubulin; WT, wild-type.
