# Supplementary material for: Loss of Lipid Carrier ApoE Exacerbates Brain Glial and Inflammatory Responses after Lysosomal GBA1 Inhibition
Source: Cells. 2023 Nov 2;12(21):2564. doi: 10.3390/cells12212564 (PMC10647680; doi:10.3390/cells12212564)
Supplement: Supplementary file 1 [file cells-12-02564-s001.zip › cells-2569986-supplementary.pdf]

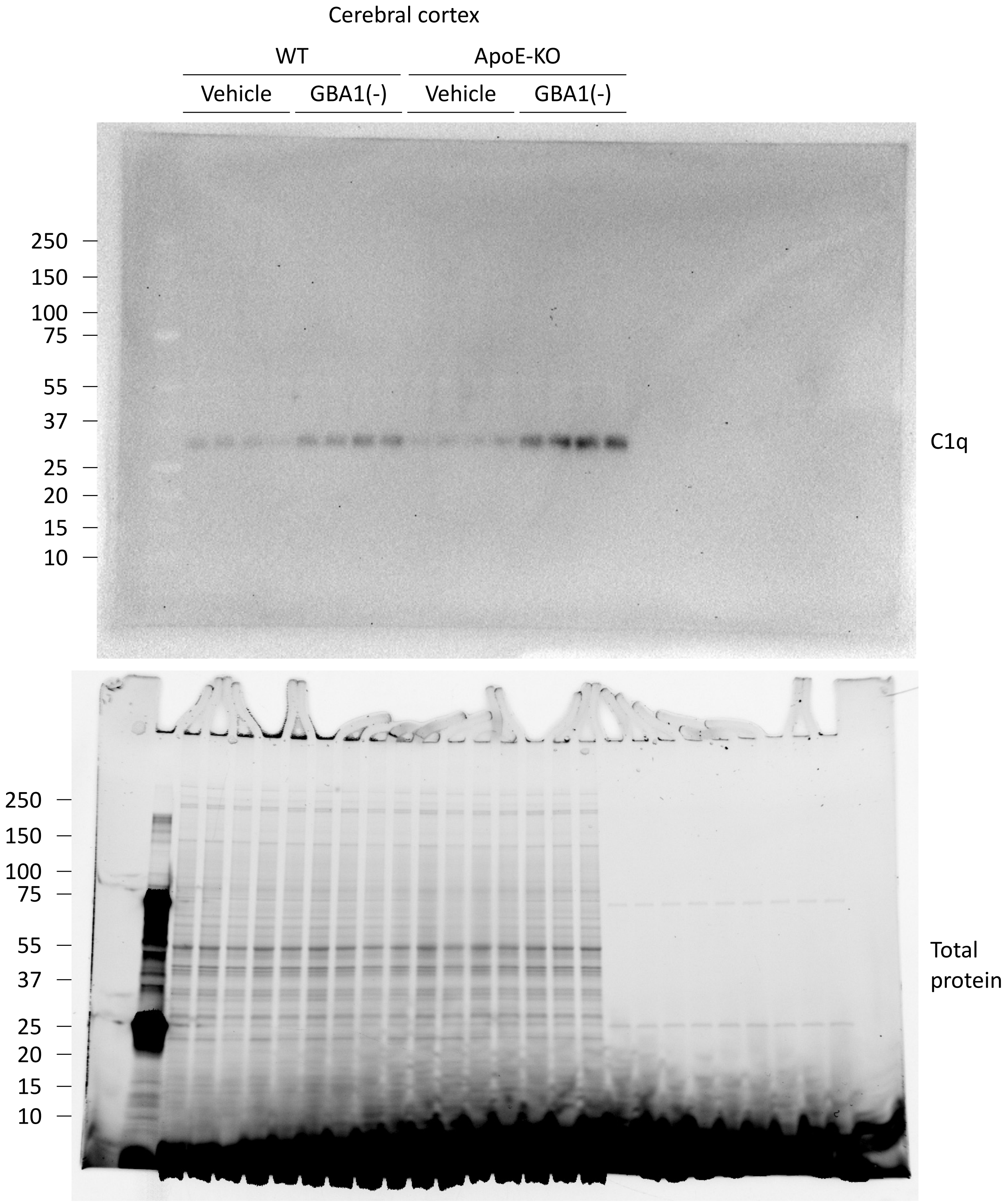

Figure S1. GBA1 inhibition increased C1q levels in the cerebral cortex and was amplified by loss of ApoE. Uncropped images of the C1q Western blot and total protein loading used to create the representative gel image in figure 3B.

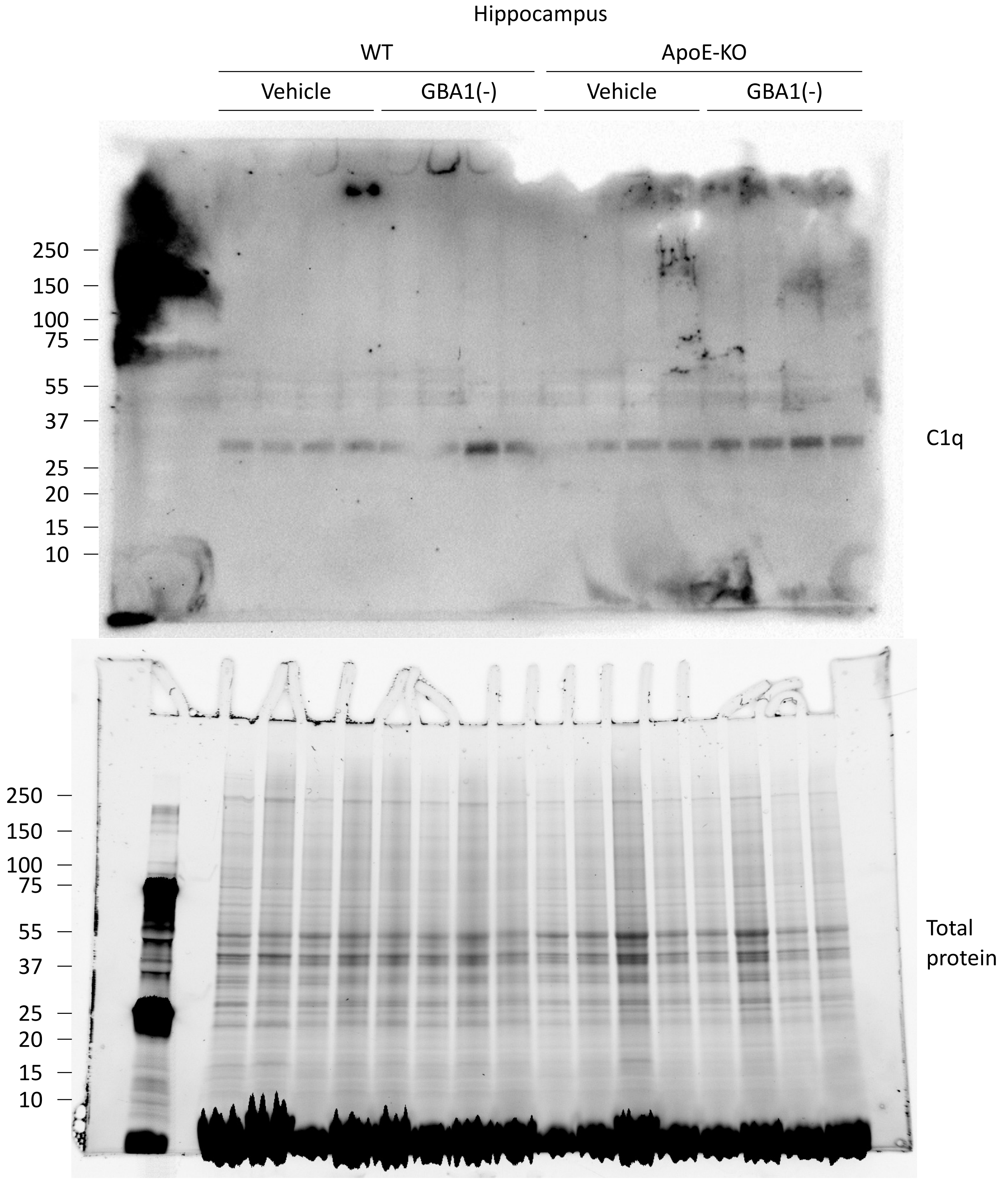

Figure S2. GBA1 inhibition increased C1q levels in the hippocampus and was amplified by loss of ApoE. Uncropped images of the C1q Western blot and total protein loading used to create the representative gel image in figure 3D.

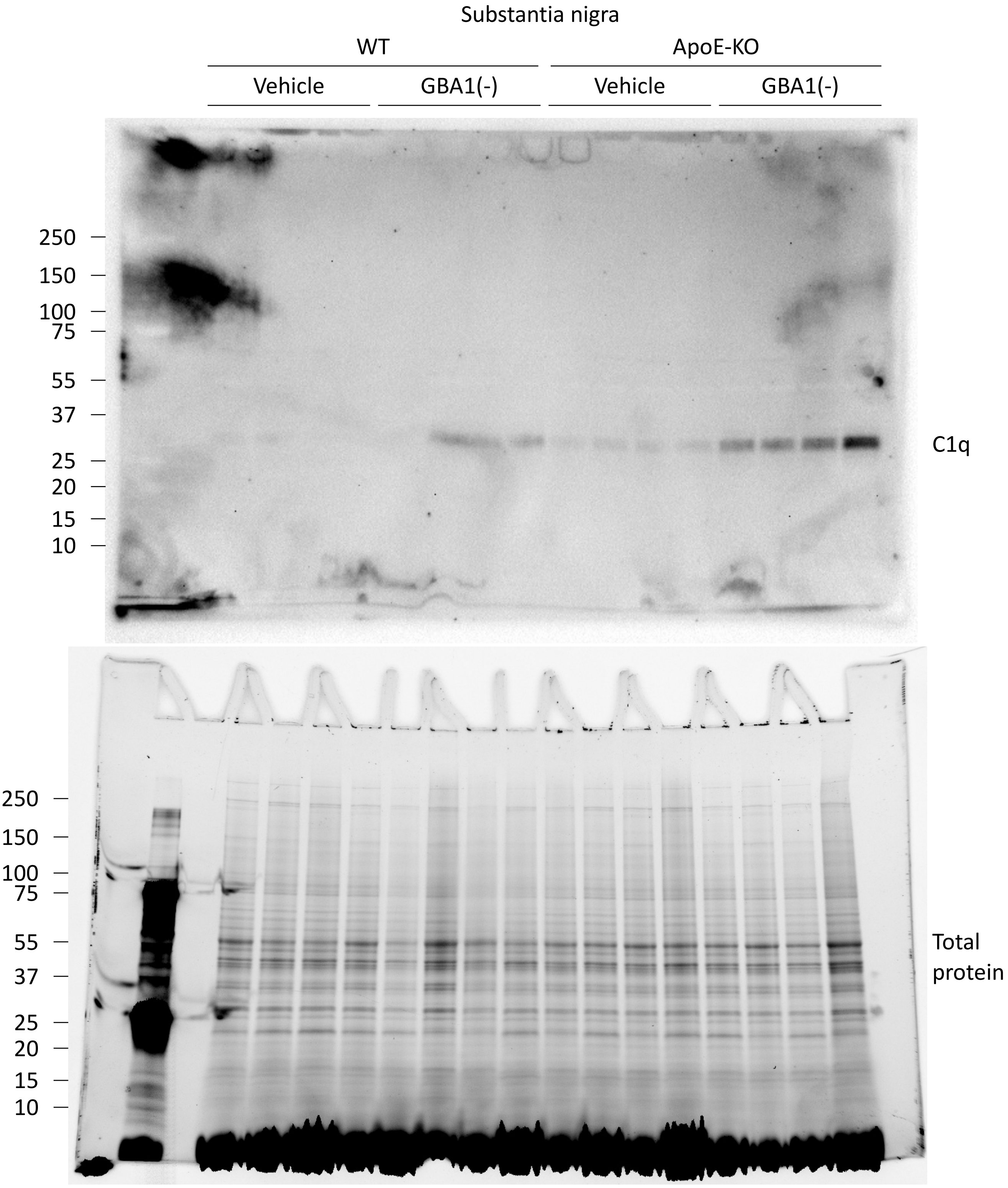

Figure S3. GBA1 inhibition increased C1q levels in the substantia nigra and was amplified by loss of ApoE. Uncropped images of the C1q Western blot and total protein loading used to create the representative gel image in figure 3F.

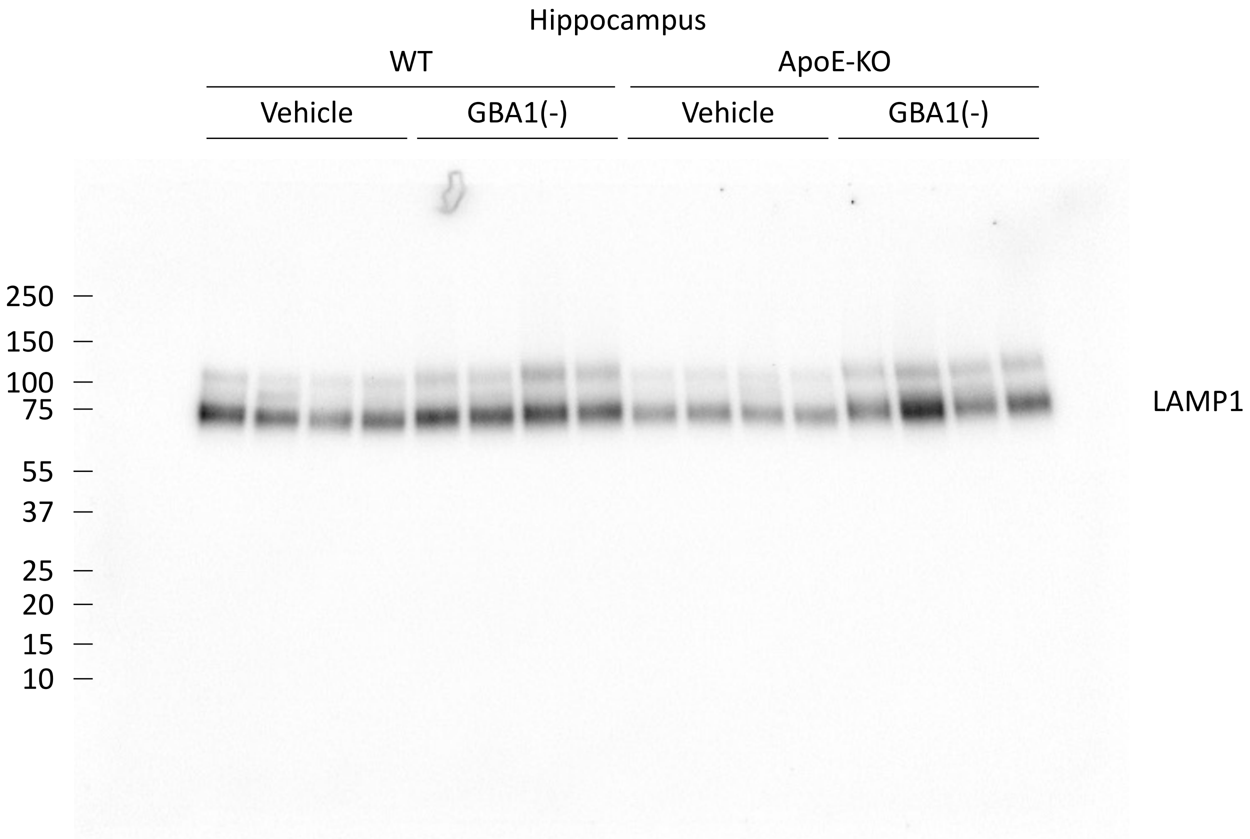

Figure S4. LAMP1 protein levels were increased after GBA1 inhibition. Uncropped image of the LAMP1 Western blot used to create the representative gel image in figure 5A.

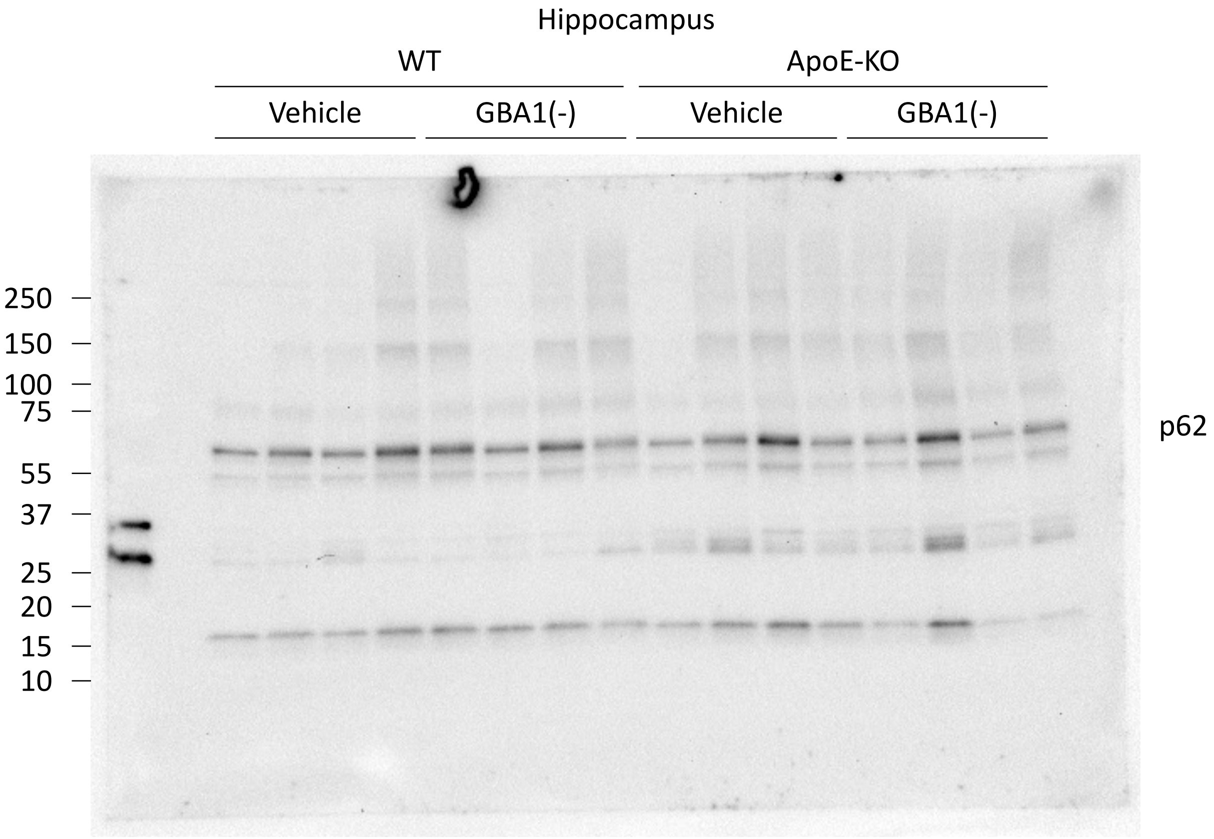

Figure S5. p62 protein levels were unchanged after GBA1 inhibition. Uncropped image of the p62 Western blot used to create the representative gel image in figure 5A.

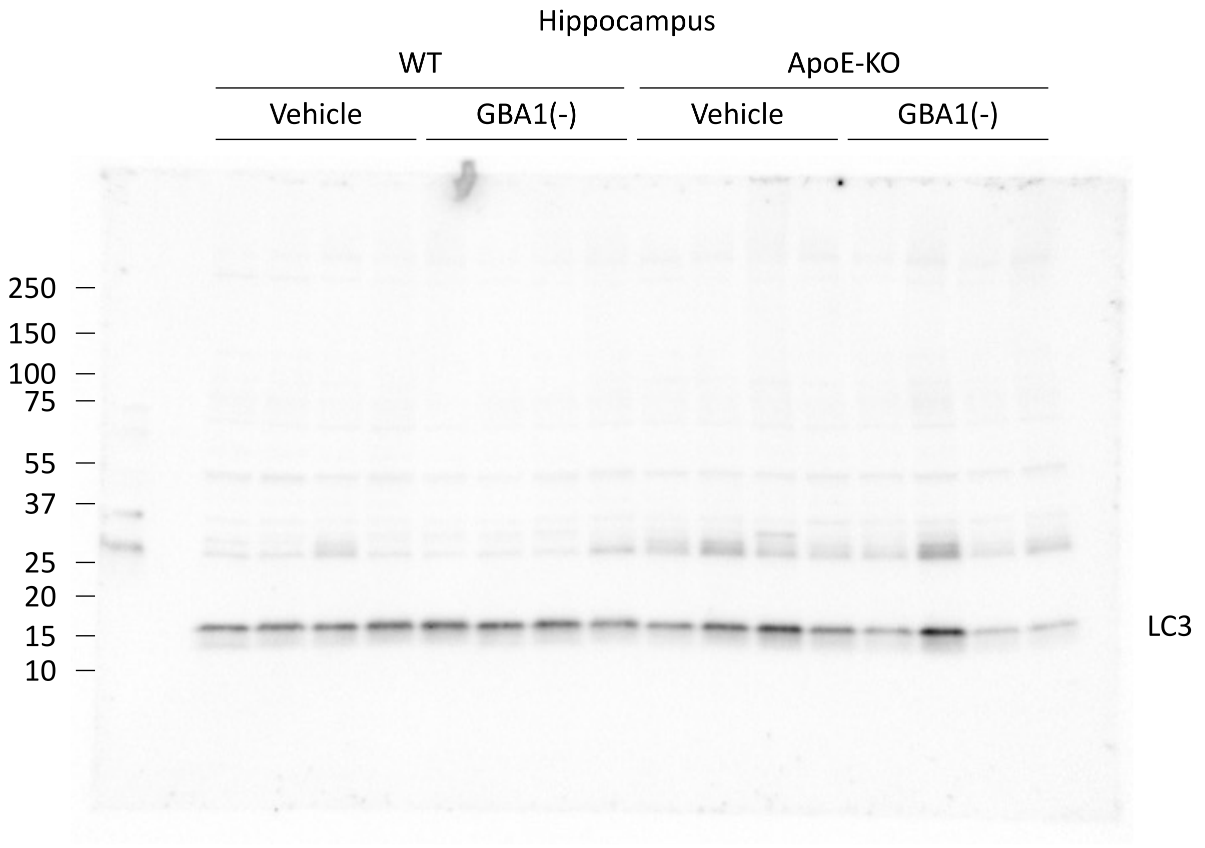

Figure S6. LC3 protein levels were unchanged after GBA1 inhibition. Uncropped image of the LC3 Western blot used to create the representative gel image in figure 5A.

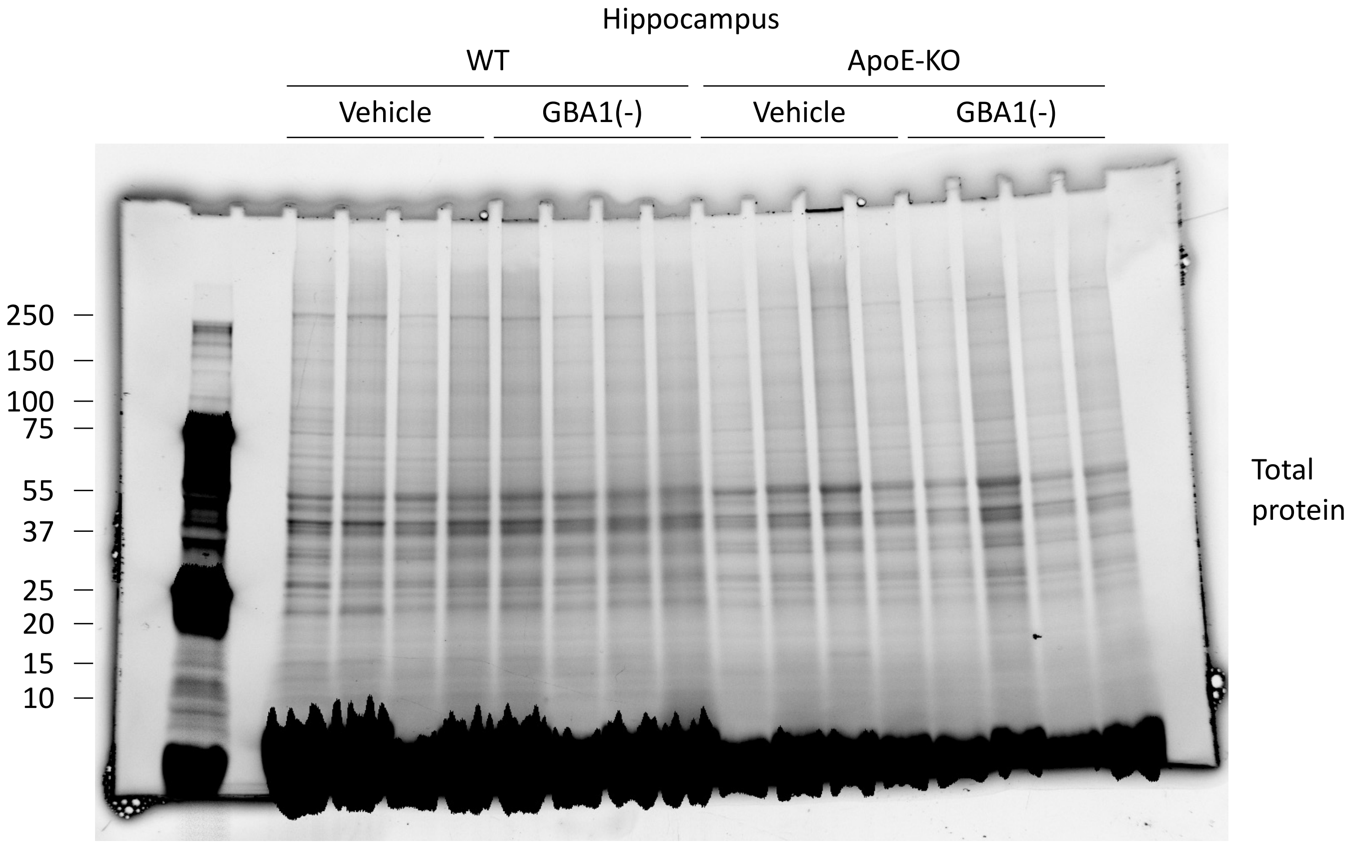

Figure S7. Total protein loading levels. Uncropped image of the protein gel showing total protein loading used to create the representative gel image in figure 5A.

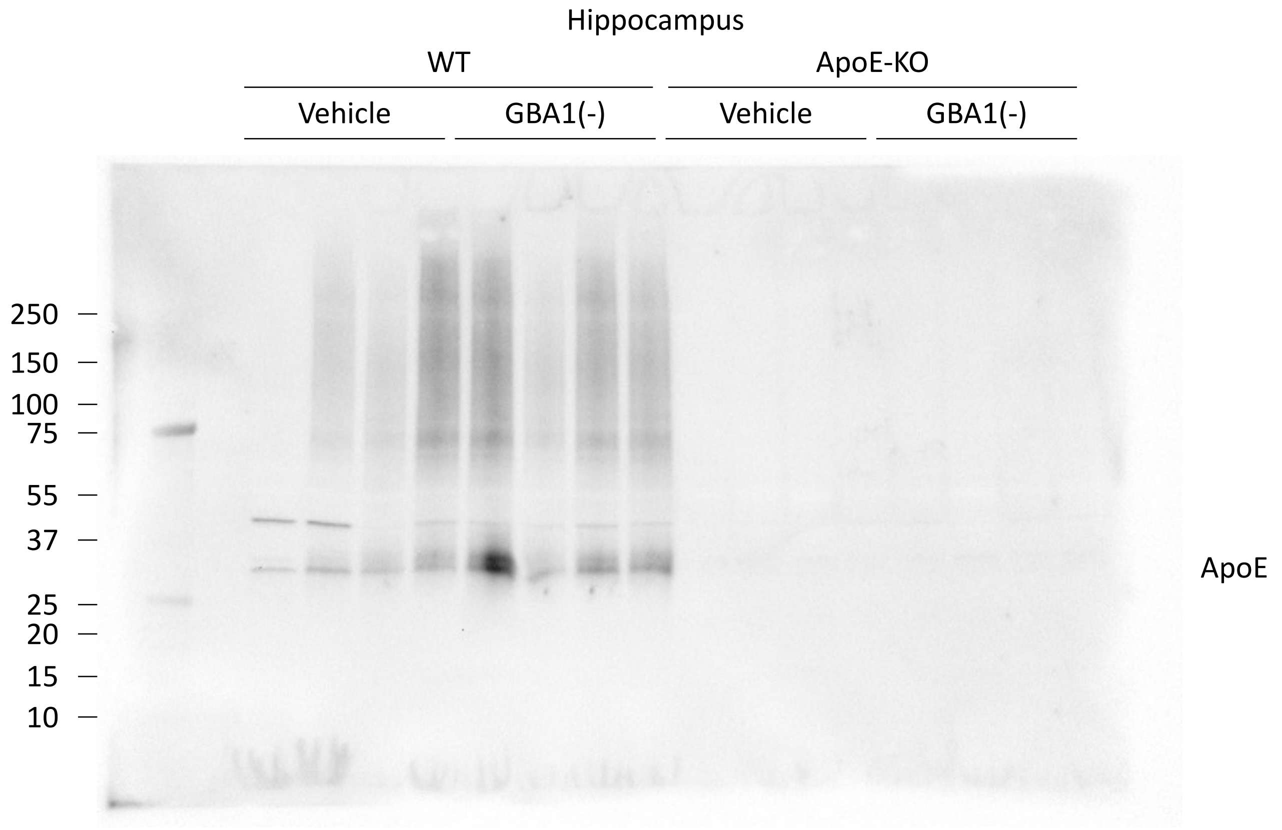

Figure S8. ApoE protein levels were increased after GBA1 inhibition. Uncropped image of the ApoE Western blot used to create the representative gel image in figure 5E.

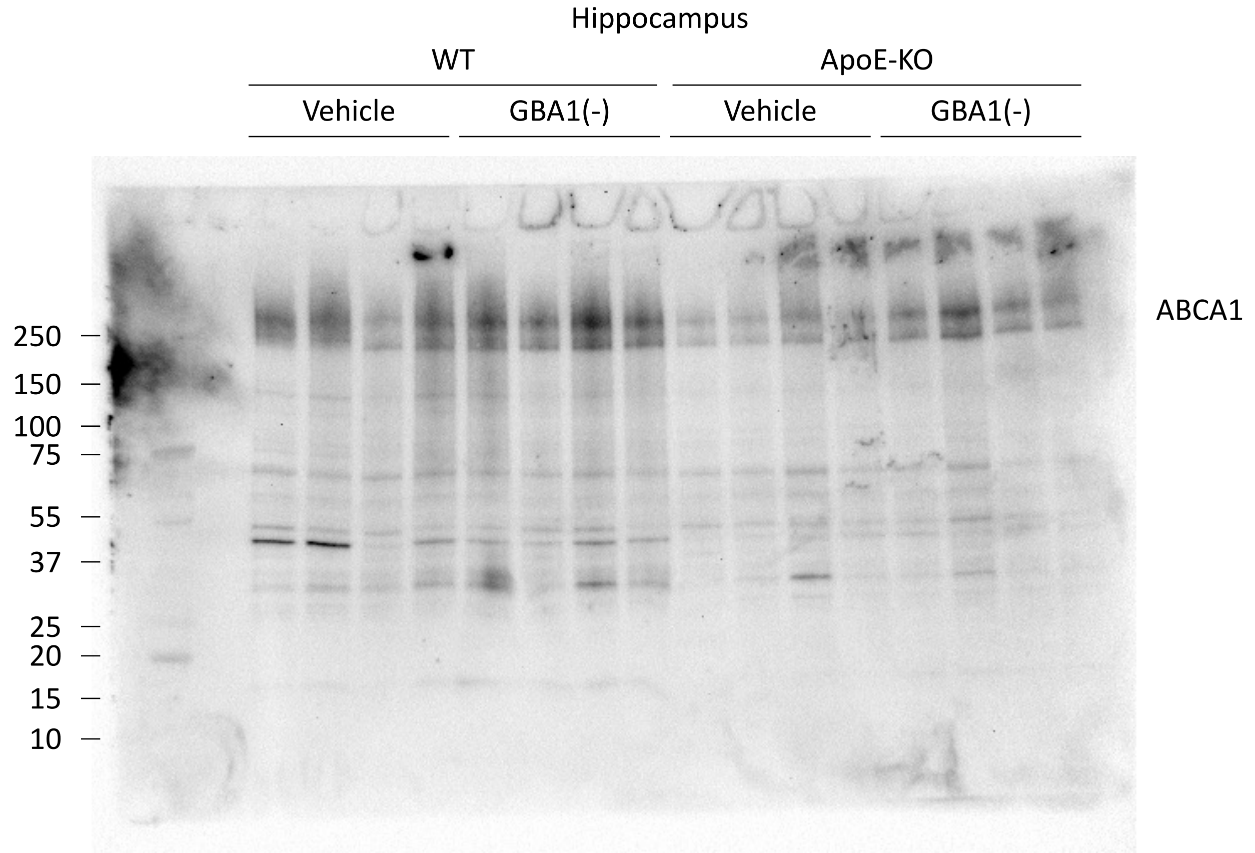

Figure S9. ABCA1 protein levels were reduced in the absence of ApoE. Uncropped image of the ABCA1 Western blot used to create the representative gel image in figure 5E.

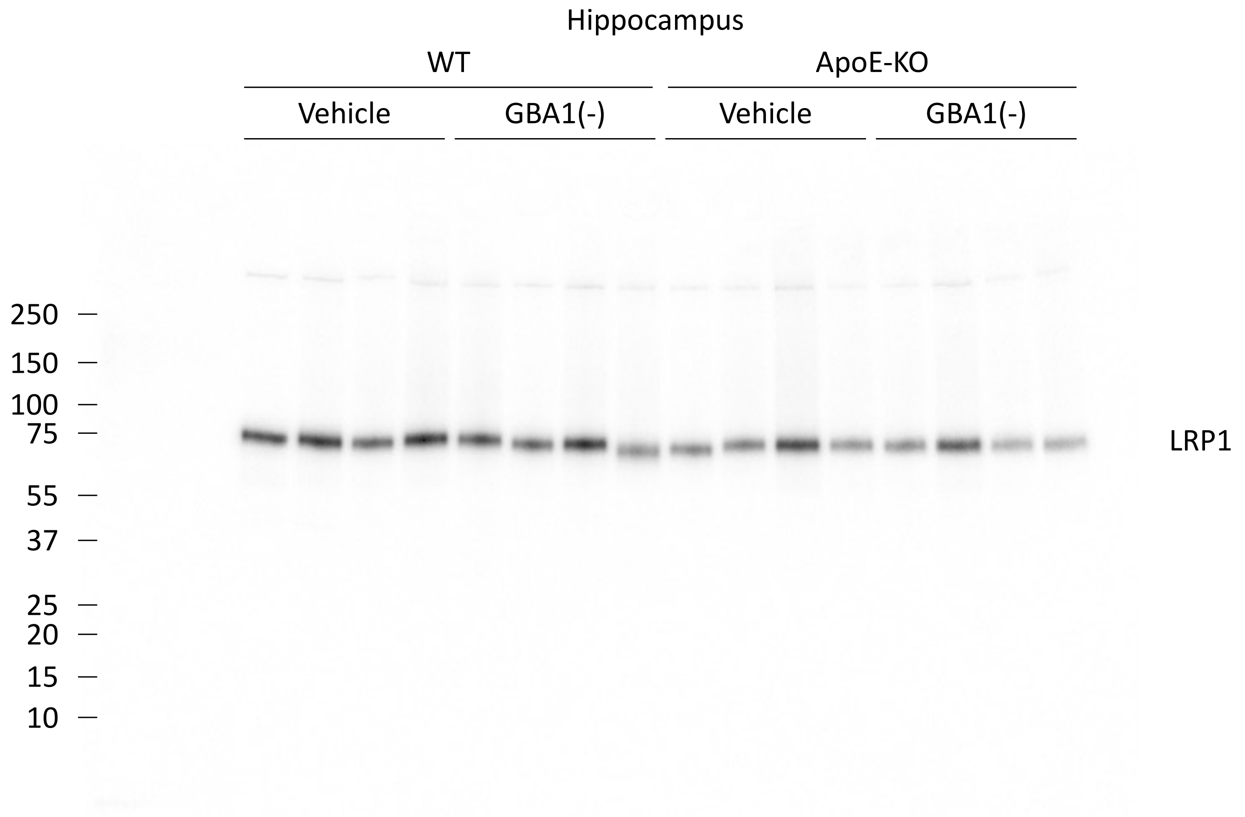

Figure S10. LRP1 protein levels were reduced in the absence of ApoE. Uncropped image of the LRP1 Western blot used to create the representative gel image in figure 5E.

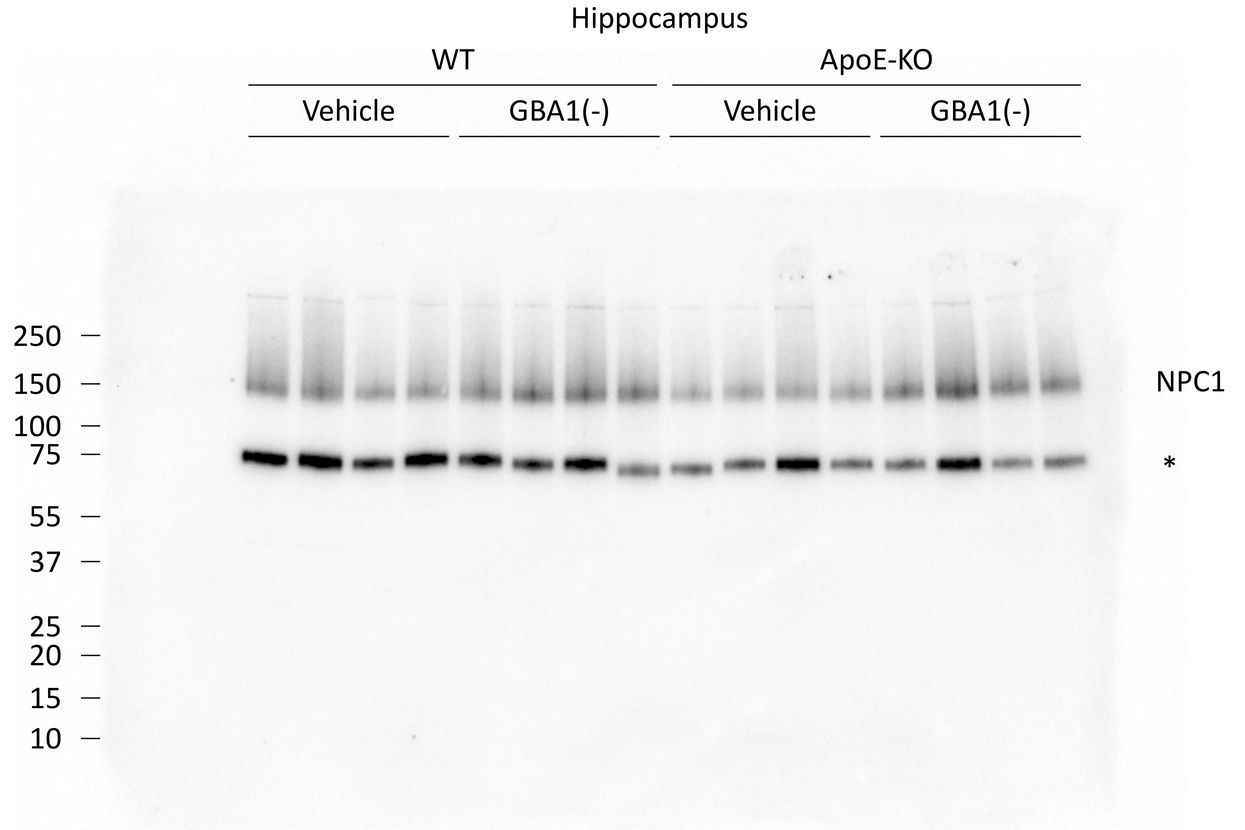

Figure S 11. NPC1 protein levels increased after GBA1 inhibition. Uncropped image of the NPC1 Western blot used to create the representative gel image in figure 5E. Asterisks annotate bands not specific to NPC1.

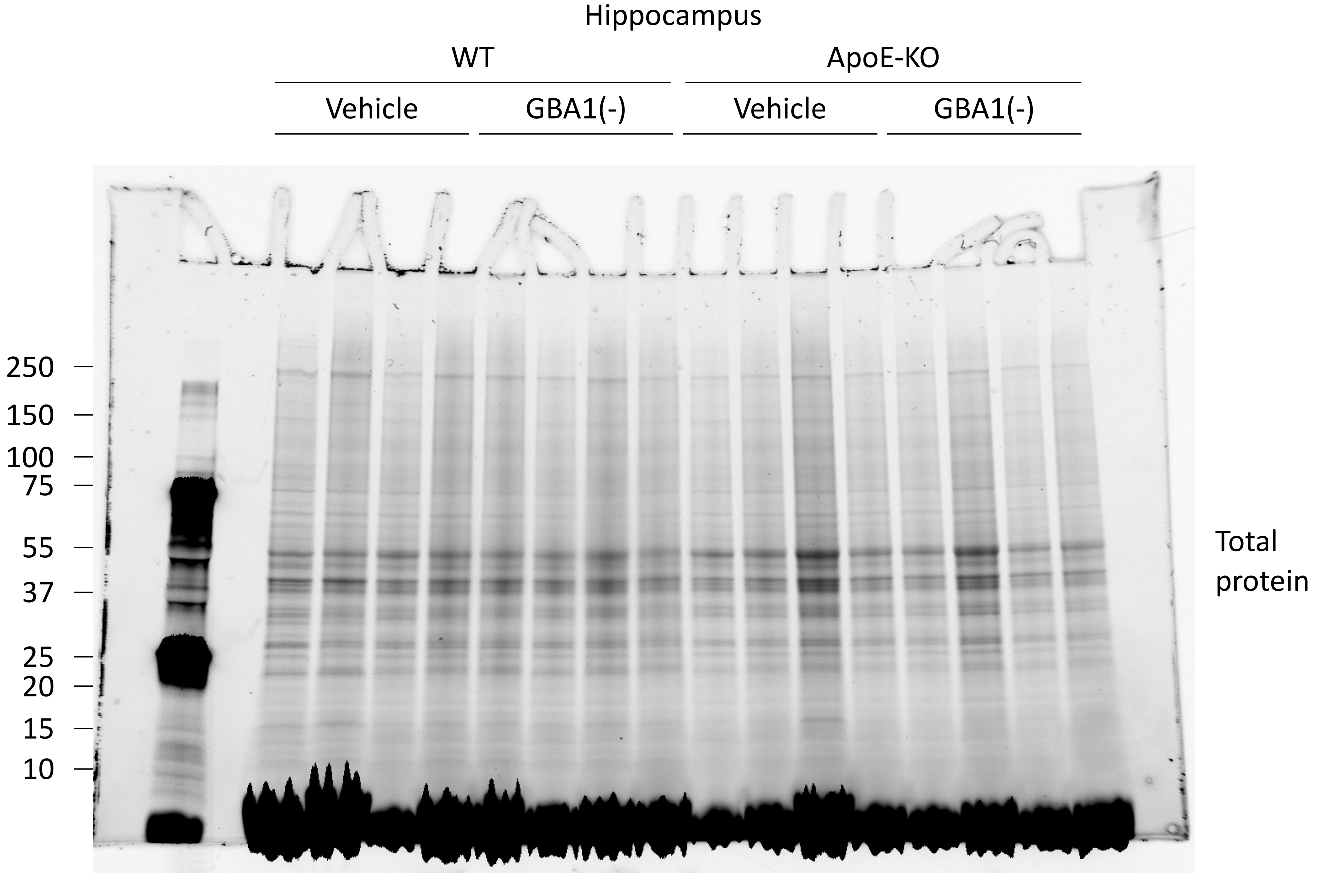

Figure S12. Total protein loading levels. Uncropped image of the protein gel showing total protein loading used to create the representative gel image in figure 5E.

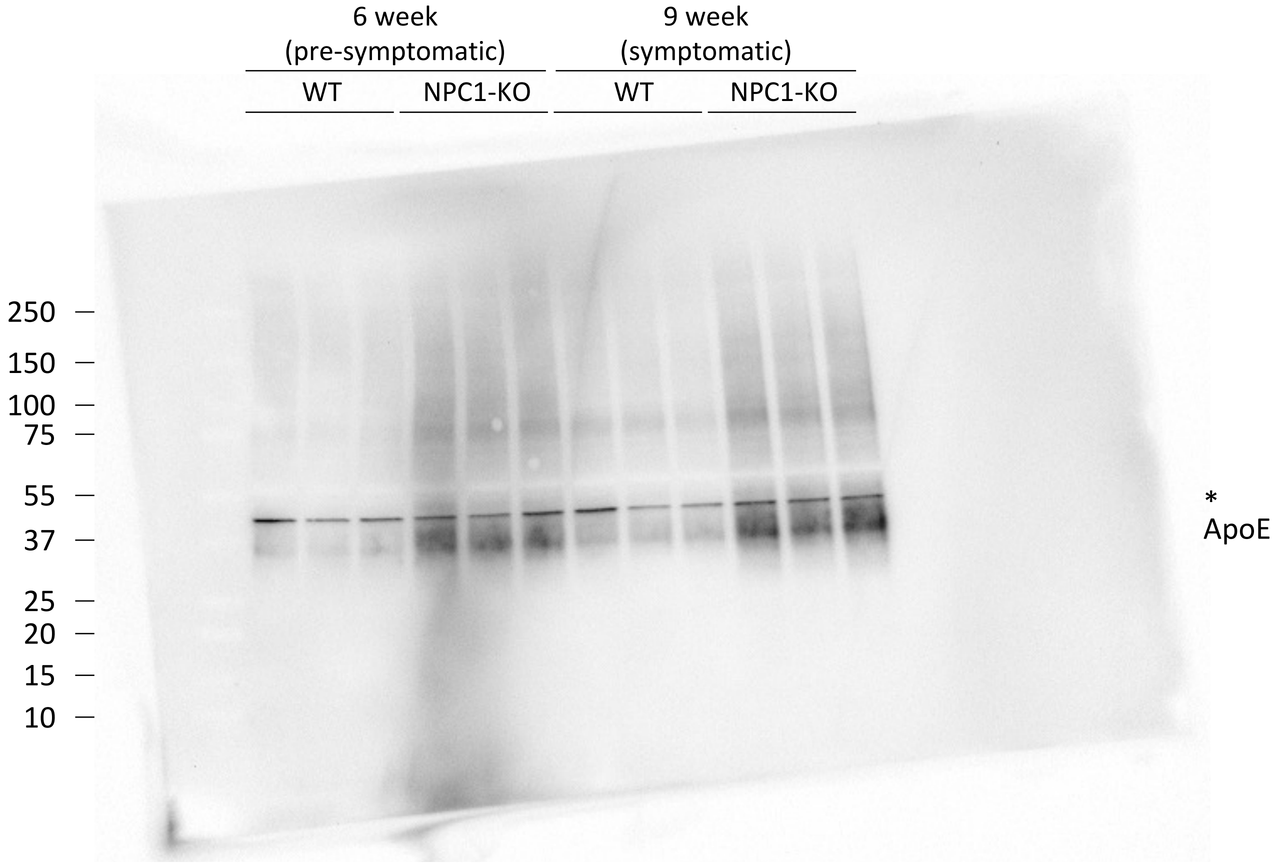

Figure S13. ApoE protein levels were elevated in mouse brains in the absence of NPC1. Uncropped image of the ApoE Western blot used to create the representative gel image in figure 6A. Asterisks annotate bands not specific to ApoE.

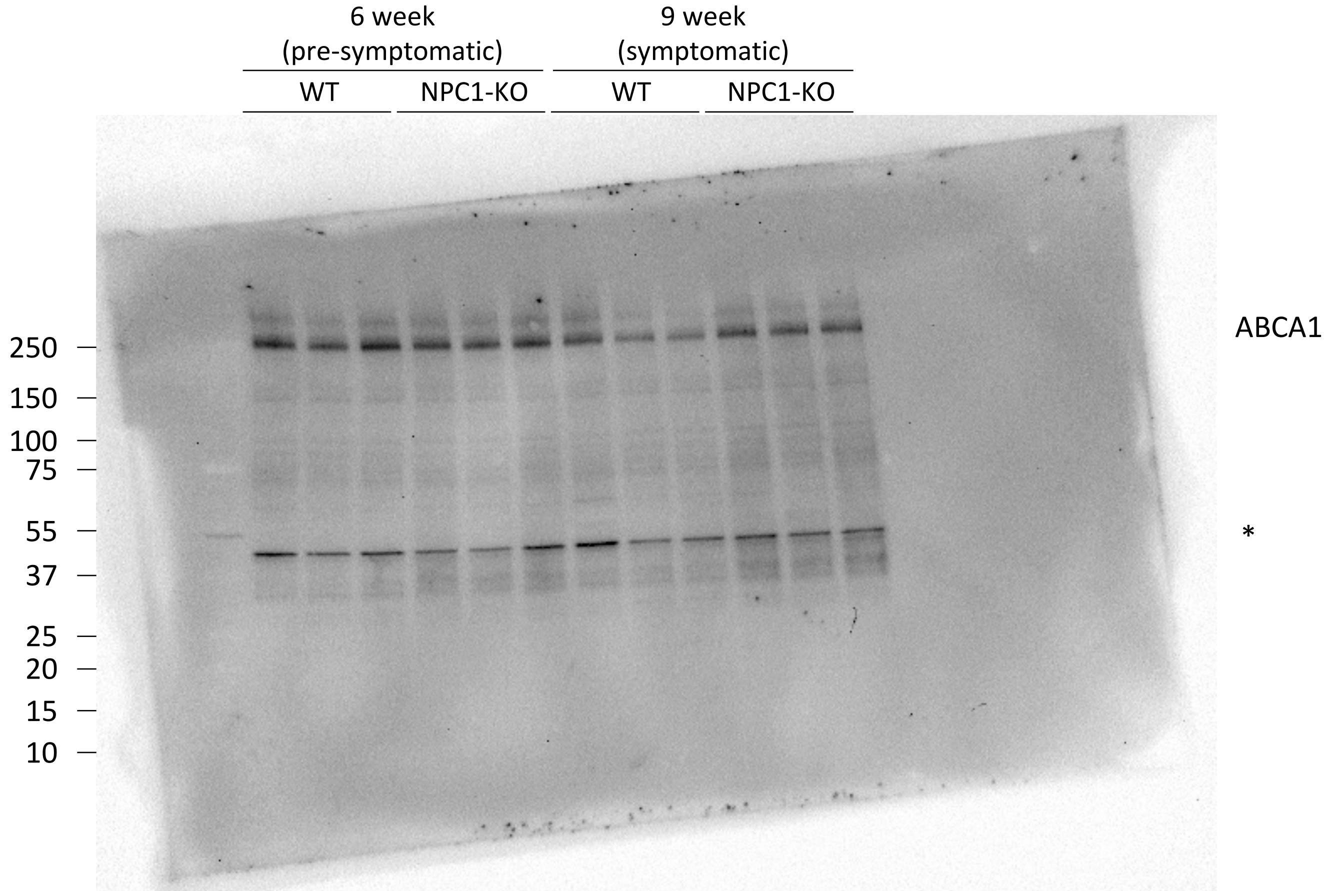

Figure S 14. ABCA1 protein levels were unchanged in mouse brains in the absence of NPC1. Uncropped image of the ApoE Western blot used to create the representative gel image in figure 6A. Asterisks annotate bands not specific to ABCA1.

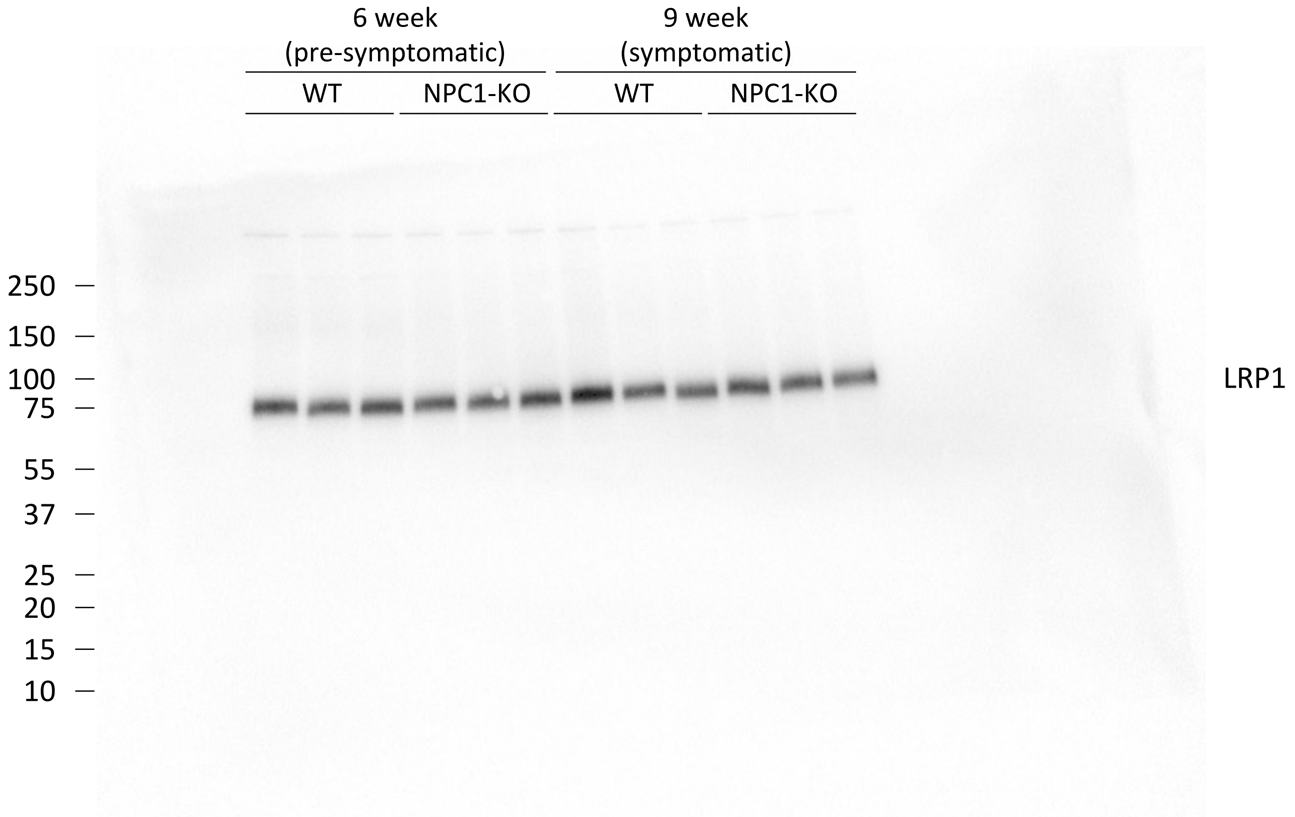

Figure S15. LRP1 protein levels were unchanged in mouse brains in the absence of NPC1. Uncropped image of the LRP1 Western blot used to create the representative gel image in figure 6A.

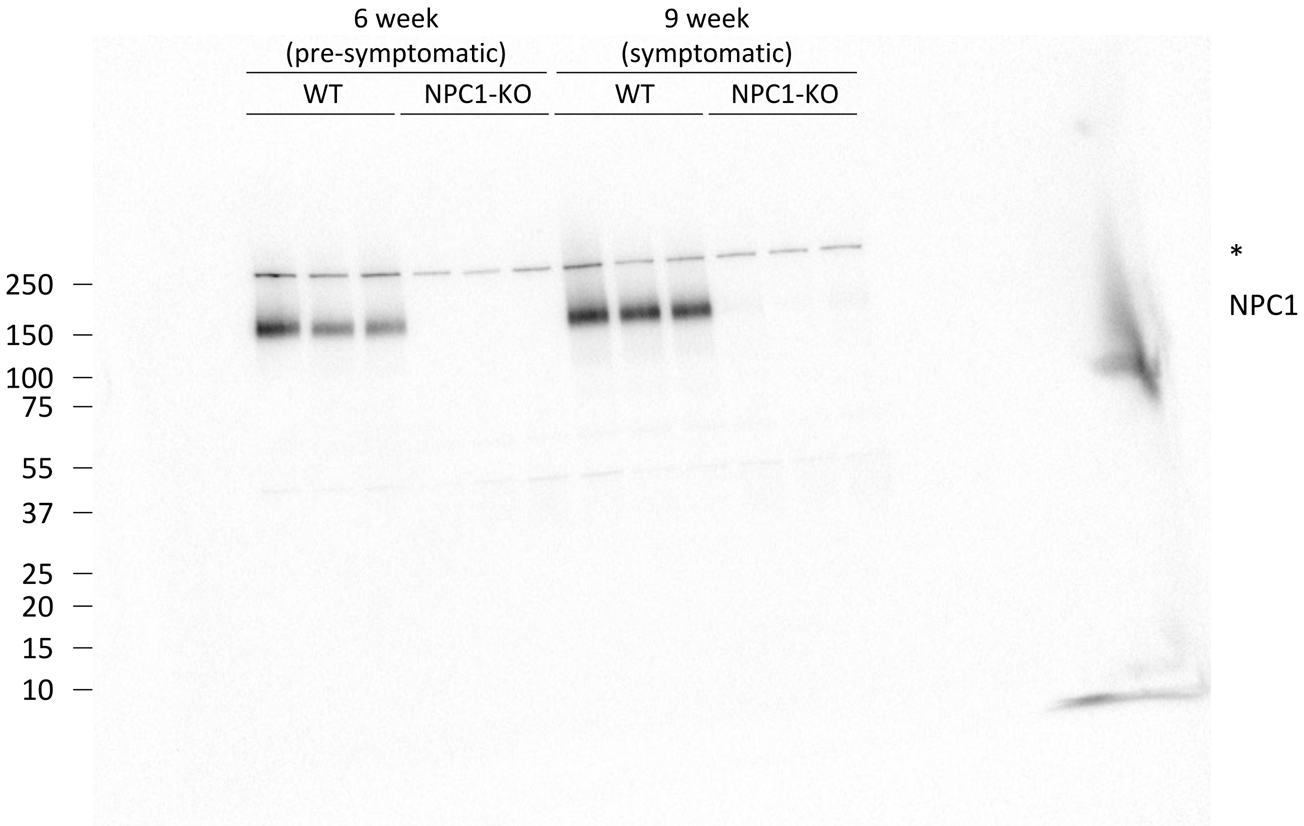

Figure S16. NPC1 protein was absent from NPC1 knockout mouse brains. Uncropped image of the NPC1 Western blot used to create the representative gel image in figure 6A. Asterisks annotate bands not specific to NPC1.

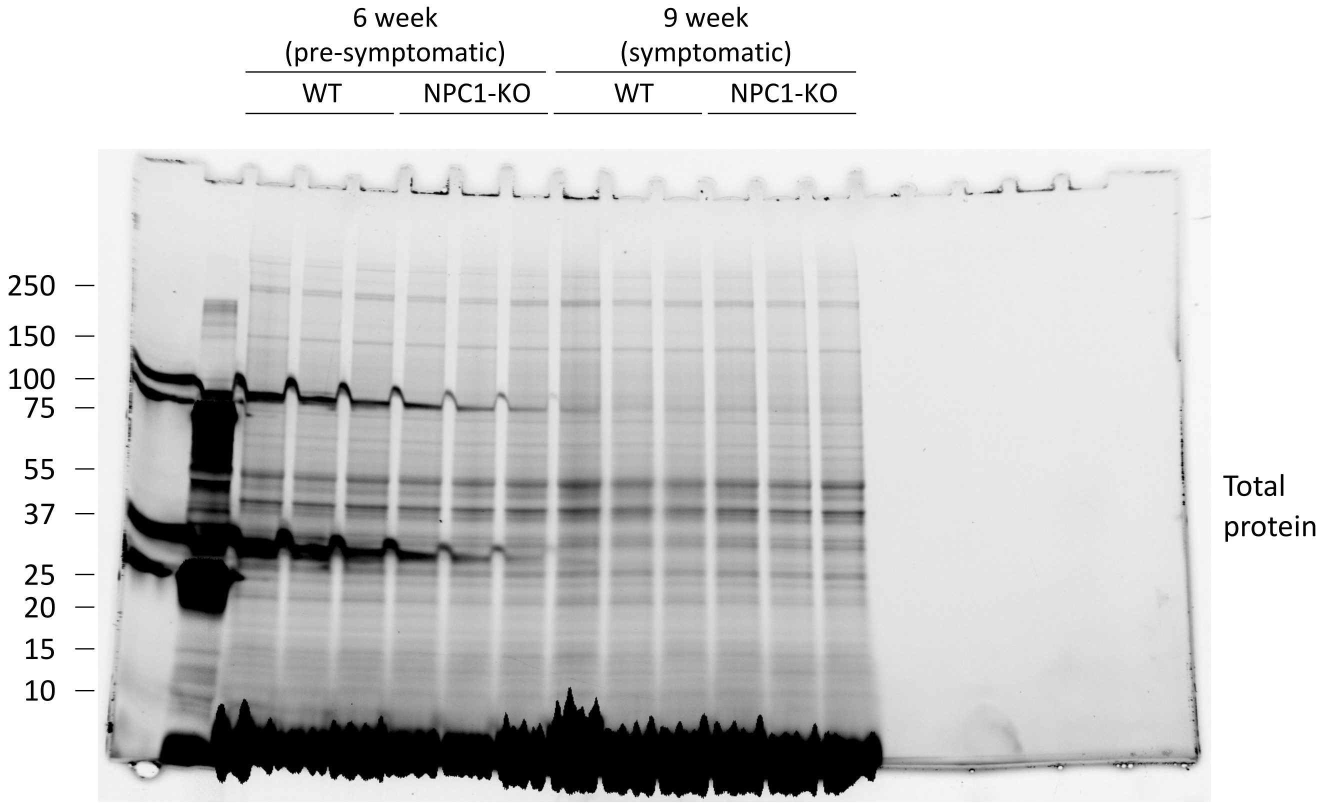

Figure S17. Total protein loading levels. Uncropped image of the protein gel showing total protein loading used to create the representative gel image in figure 6A.

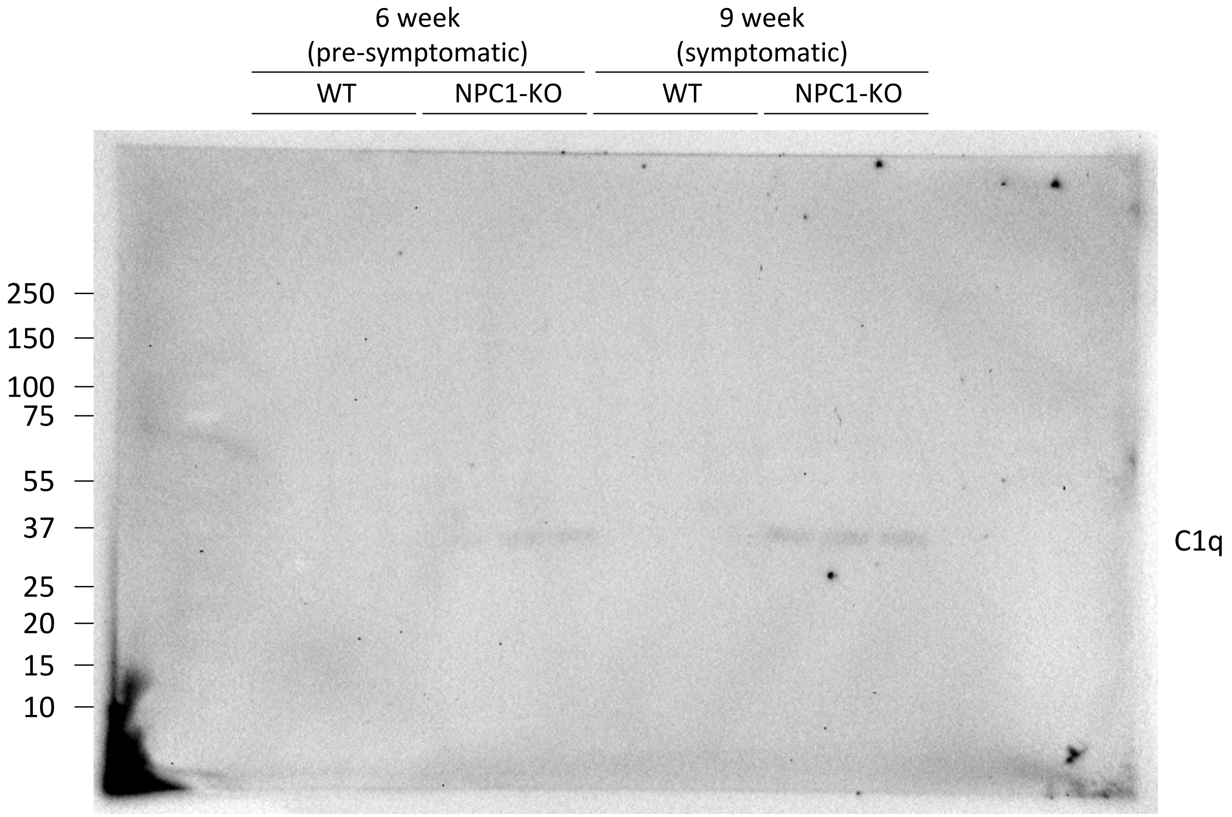

Figure S18. C1q protein levels were increased in mouse brains in the absence of NPC1. Uncropped image of the C1q Western blot used to create the representative gel image in figure 6F.

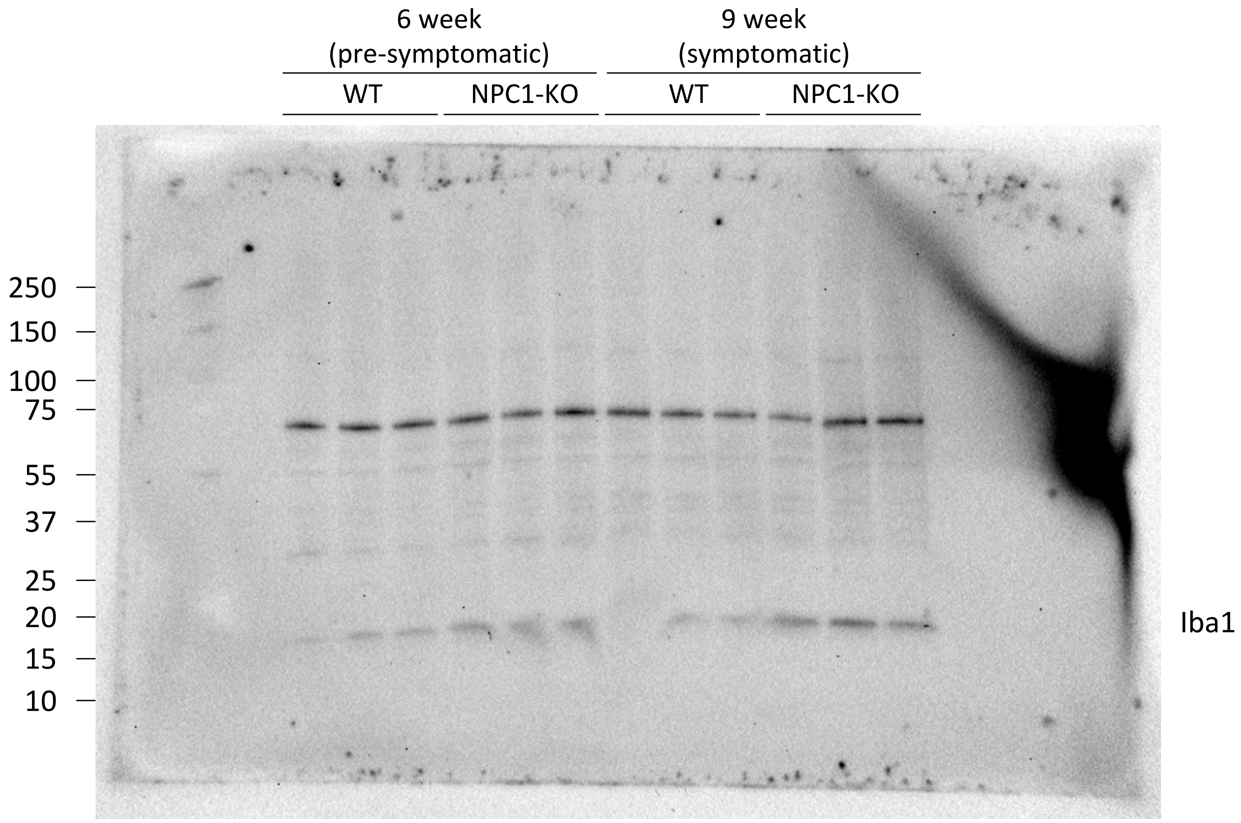

Figure S19. Iba1 protein levels were increased in mouse brains in the absence of NPC1. Uncropped image of the Iba1 Western blot used to create the representative gel image in figure 6F.

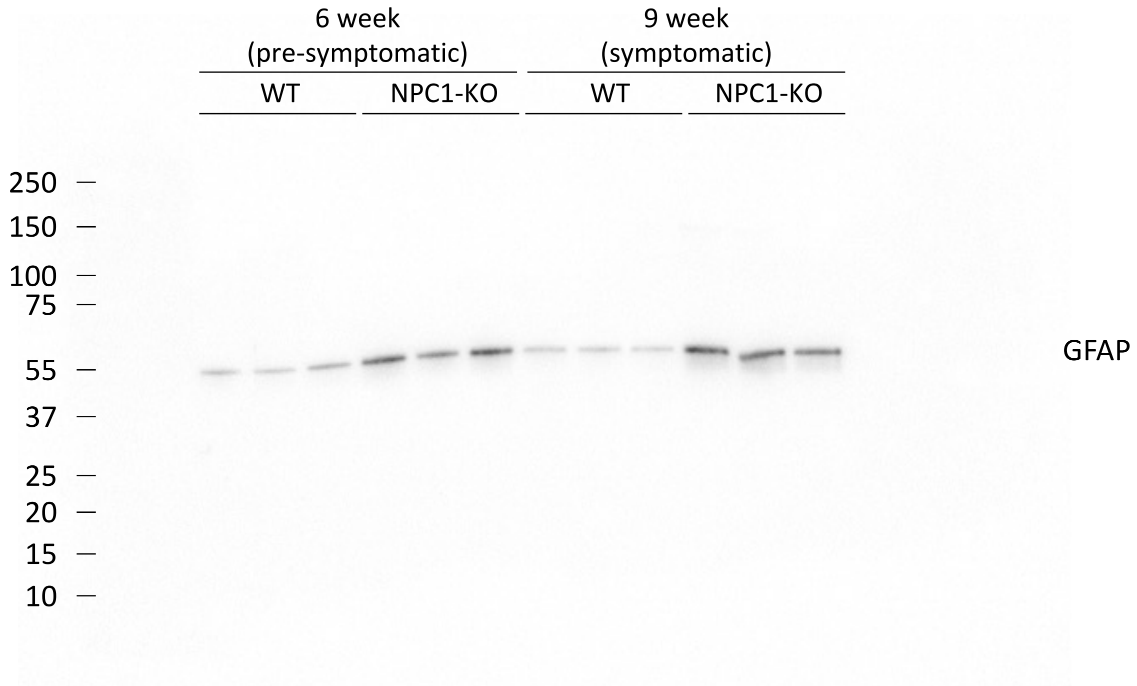

Figure S20. GFAP protein levels were increased in mouse brains in the absence of NPC1. Uncropped image of the GFAP Western blot used to create the representative gel image in figure 6F.

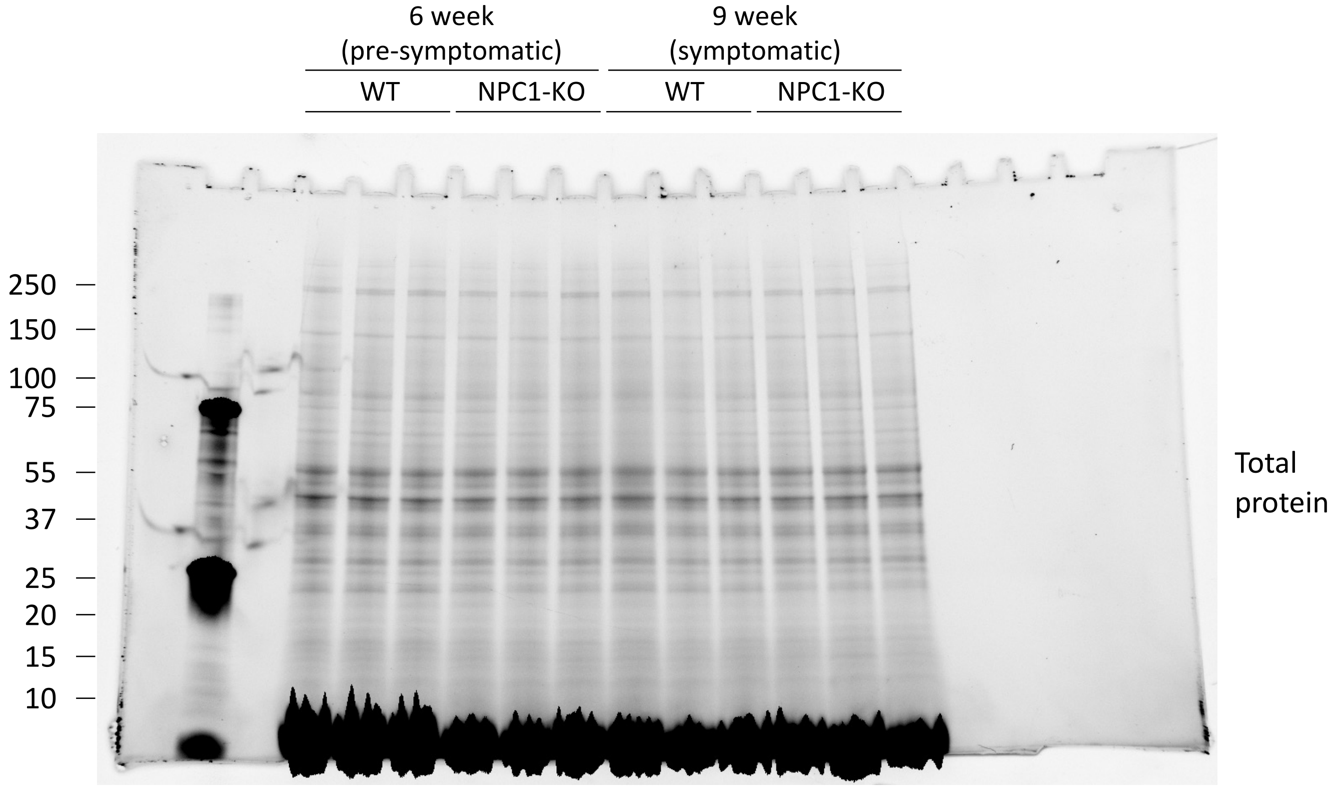

Figure S21. Total protein loading levels. Uncropped image of the protein gel showing total protein loading used to create the representative gel image in figure 6F.
